# A Novel Bipartite Centrosome Coordinates the Apicomplexan Cell Cycle

**DOI:** 10.1371/journal.pbio.1002093

**Published:** 2015-03-03

**Authors:** Elena S. Suvorova, Maria Francia, Boris Striepen, Michael W. White

**Affiliations:** 1 Departments of Molecular Medicine & Global Health and the Florida Center for Drug Discovery and Innovation, University of South Florida, Tampa, Florida, United States of America; 2 Center for Tropical and Emerging Global Diseases and Department of Cellular Biology, University of Georgia, Athens, Georgia, United States of America; Dana-Farber Cancer Institute, UNITED STATES

## Abstract

Apicomplexan parasites can change fundamental features of cell division during their life cycles, suspending cytokinesis when needed and changing proliferative scale in different hosts and tissues. The structural and molecular basis for this remarkable cell cycle flexibility is not fully understood, although the centrosome serves a key role in determining when and how much replication will occur. Here we describe the discovery of multiple replicating core complexes with distinct protein composition and function in the centrosome of *Toxoplasma gondii*. An outer core complex distal from the nucleus contains the TgCentrin1/TgSfi1 protein pair, along with the cartwheel protein TgSas-6 and a novel Aurora-related kinase, while an inner core closely aligned with the unique spindle pole (centrocone) holds distant orthologs of the CEP250/C-Nap protein family. This outer/inner spatial relationship of centrosome cores is maintained throughout the cell cycle. When in metaphase, the duplicated cores align to opposite sides of the kinetochores in a linear array. As parasites transition into S phase, the cores sequentially duplicate, outer core first and inner core second, ensuring that each daughter parasite inherits one copy of each type of centrosome core. A key serine/threonine kinase distantly related to the MAPK family is localized to the centrosome, where it restricts core duplication to once per cycle and ensures the proper formation of new daughter parasites. Genetic analysis of the outer core in a temperature-sensitive mutant demonstrated this core functions primarily in cytokinesis. An inhibition of ts-TgSfi1 function at high temperature caused the loss of outer cores and a severe block to budding, while at the same time the inner core amplified along with the unique spindle pole, indicating the inner core and spindle pole are independent and co-regulated. The discovery of a novel bipartite organization in the parasite centrosome that segregates the functions of karyokinesis and cytokinesis provides an explanation for how cell cycle flexibility is achieved in apicomplexan life cycles.

## Abstract 2

The apicomplexan parasite *Toxoplasma gondii* has a unique centrosome with two specialized compartments, potentially explaining the remarkable flexibility in life cycle that these organisms can show in diverse host cells.

## Author Summary

Apicomplexan parasites infect many different hosts and tissues, causing numerous human diseases, including malaria. These important pathogens have a peculiar cell cycle in which chromosomes sometimes amplify to remarkable levels, followed by concerted cell division—providing an unusual proliferative capacity. This capacity for proliferation, combined with an ability to change the scale of replication when needed, are hallmarks of the cell cycles of these parasites. Yet the molecular mechanism responsible for these peculiar cell cycles remains one of the unsolved mysteries of Apicomplexa biology. Here we show that the centrosome—an organelle that orchestrates several aspects of the cell cycle—of the apicomplexan parasite *Toxoplasma gondii* contains specialized structures that coordinate parasite cell division. Our findings demonstrate that a two-part centrosomal architecture, comprising an inner and an outer core with distinct protein compositions, segregates the processes of mitosis from the assembly of new daughter parasites. The modular organization of the centrosome offers an explanation for how cell division can be suspended while the parasites amplify their genome to the biotic scale required for their life cycles. It is unknown whether these distinct centrosome core complexes evolved independently in Apicompexa. Another possibility is that the foundations for these mechanisms were present in the original eukaryote, which could explain how the distinct extranuclear centrosome of animal cells and the novel yeast spindle pole body of the nuclear envelope may have evolved from a common ancestor.

## Introduction

Infection with apicomplexan parasites is the cause of numerous important human diseases, including malaria, cryptosporidiosis, and toxoplasmosis. Pathogenesis of these diseases is closely tied to parasite replication [1] and the destruction of host cells, leading to tissue and organ damage. This fundamental relationship between parasite growth and disease is evident by the action of drugs used to combat these infections since the best treatments all reduce or block parasite proliferation. Existing therapies, in particular for malaria, are under constant pressure from acquired parasite drug resistance, a situation that requires a broad portfolio of antiparasitic compounds with different parasite-specific targets. The peculiar proliferative cycles of Apicomplexa parasites differ substantially from the hosts they inhabit and should offer fertile ground to supply an active pipeline of new treatments. To fulfill this promise, we need a better understanding of the unique structural and molecular features of parasite proliferation.

Modern Apicomplexa are the result of millions of years of evolution [2], involving successful encounters with many invertebrate and vertebrate hosts that have led to an extraordinary worldwide distribution. The development of specialized invasion and replication strategies [3–5] has permitted these parasites to surmount a variety of host-defensive barriers and achieve sufficient expansion in many different host tissues. Apicomplexan replication has adapted to different host cells, most commonly using a sequence of two chromosome replication cycles uniquely regulated in different parasite genetic lineages [4]. A single G1 phase that varies in length with the scale of parasite production precedes a first chromosome cycle (S/M_n_), the biosynthetic focus of which is genome replication (nuclear cycle), followed by a unconventional chromosome cycle (S/M_n+1_) that produces infectious parasites (budding cycle). The budding cycle is restricted to a single round of chromosome replication, and therefore, the amplification of the genome in the nuclear cycle determines the scale of biotic expansion. That scale can range depending on the species from a few to thousands of parasites produced from a single infected cell. Through simple variation in the nuclear to budding cycle sequence, apicomplexan parasites have solved the problem of adjusting proliferation to a wide variety of host cells. What is not understood are the mechanistic details that afford this tremendous cell division flexibility, while also preserving the fidelity of chromosome replication. Viewed from the restricted principles of model eukaryotic cell cycles, successful Apicomplexa replication often appears chaotic and in violation of some basic cell cycle restrictions (e.g. “copy once only once” in the nuclear cycle). This paradox is one of the major mysteries of the phylum Apicomplexa.

During their life cycle, *Toxoplasma gondii* parasites switch between multi- (merozoite stage) and binate-nuclear replication (tachyzoite stage) [6], with the binary division cycle of the tachyzoite (called endodyogeny, i.e. “inside two are borne”) now a major experimental model for understanding basic principles of apicomplexan proliferation. The cell cycle periods of the tachyzoite [4,7–10] are reasonably defined and have provided evidence for major checkpoint control that was exploited to synchronize parasite growth [9,11,12]. The most unusual feature of Apicomplexa cell division is budding, which occurs by the assembly of daughter cells within or from the mother cell using a highly ordered process [3] that is accompanied by the de novo synthesis and packaging of invasion organelles [13]. In *T*. *gondii* tachyzoites, as in *Plasmodium falciparum* merozoites, assembly of new parasites (budding) is guided by a unique cell cycle transcriptome that delivers proteins in a "just-in-time" order [7,14]. A major conclusion from these studies is that the tachyzoite centrosome has a vital role in coordinating budding and mitotic events. The centrosome is one of two sites of microtubule nucleation in the tachyzoite with direct responsibility for assembly of the intranuclear spindle. A second microtubule-organizing center (MTOC) is the polar ring of the apical complex. This MTOC organizes the subpellicular microtubules (MTs) scaffold supporting the pellicle that gives the parasite its shape [3]. Importantly, the centrosome also governs the position and activity of this second MTOC through a physical tether, the striated fiber [15]. During mitosis in tachyzoites, a polar striated fiber emerges from each centrosome and at its other end gives rise to the MTOC that defines the daughter cells. Both centers are thus aligned and physically connected [15].

Across the Apicomplexa phylum, similar structural and mitotic principles are observed to govern parasite division. The centrosome (also called a centriolar plaque) has a central role in regulating the Apicomplexa cell cycle [4]. Ultrastructural studies of different coccidian parasites have demonstrated that the duplication of the centrosome occurs prior to budding, and this involves an unusual parallel configuration of the internal centriole structures [4,16–18]. Assembly of new daughters initiates in close proximity to these structures [8,19,20], and in *Toxoplasma* mutants that fail to duplicate the centrosome parasite, budding is inhibited [21–23]. Perinuclear centrosome structures also duplicate prior to each nuclear division during the intraerythocytic cycle of *P*. *falciparum* merozoites, with coordination of these structures in the last nuclear division that precedes budding [1]. A fixed spatial orientation of the centrosome to a unique protein complex embedded in the nuclear envelope, called the centrocone, is also a common structural feature of the apicomplexan mitosis [24]. Importantly, these mitotic structures are positioned near where centromeres tether chromosomes during interphase [3,4,25], and it is through the centrocone structure that spindle fibers pass into the nucleus to segregate chromosomes during mitosis [4]. The centrosome also has a master function in limiting chromosome replication in the tachyzoite that appears to require physical contact. Evidence from tachyzoite growth mutants indicates that "copy once" restrictions in the budding cycle require a connection between the centrosome and new daughter cytoskeleton [26,27]. Breaking this contact by drug treatment [28] or by genetic ablation of centrosome factors [15,22,26] leads to unregulated nuclear re-duplication and abnormal budding. To help explain the master functions associated with this mitotic organelle, we describe here the discovery of a complex internal structure of the *T*. *gondii* centrosome that is comprised of two independent and replicating core structures. The centrosome cores have unique structural and regulatory protein composition, and each core has a fixed spatial orientation to the budding and mitotic machineries. Genetic analysis of specific centrosome proteins indicates that each type of core serves distinct roles in regulating either cytokinesis or karyokinesis. Together, these results support a model whereby differential modulation of the centrosome cores could provide the flexibility required to achieve different modes of apicomplexan parasite replication.

## Results

### *Toxoplasma* Centrosome Contains Conserved and Novel Factors

**Fig 1:**
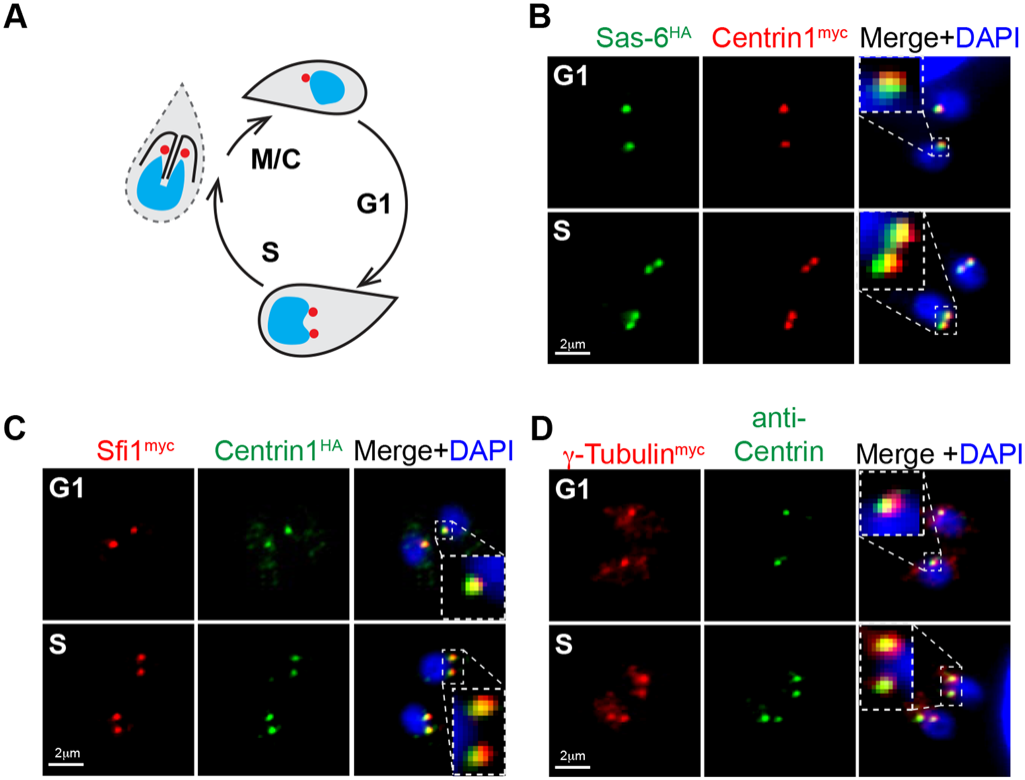
Conserved centrosomal proteins co-localize with TgCentrin1 in tachyzoites. (A) Schematic representation of the *T*. *gondii* tachyzoite centrosome cycle. Centrosome duplication occurs at the G1/S transition, and at the completion of mitosis and budding (M/C) each new daughter parasite inherits a single TgCentrin1-containing centrosome, indicated by red dot. (B) A transgenic strain expressing epitope-tagged TgSas-6^HA^ and TgCentrin1^myc^ produced by sequential genetic knock-in shows tight co-localization of these centrosome proteins. A single TgSas-6^HA^/TgCentrin1^myc^ structure in G1 phase duplicated in parasites that had entered S phase. Cell cycle phases of individual parasites and vacuoles were determined based on well-established nuclear and cell morphological criteria [4]. (C and D) Using a similar knock-in strategy, *T*. *gondii* orthologs of Sfi1 and γ-Tubulin were epitope-tagged with 3xmyc in the genomic locus. Localization of these proteins in the centrosome was established by co-staining the γ-Tubulin^myc^ transgenic strain with anti-Centrin antibody, and in the TgSfi1^myc^ transgenic clone, epitope-tagging of TgCentrin1 with 3xHA by genetic knock-in was used. DAPI co-staining (blue) was used to visualize nucleus. Magnified inset images inside the merged images highlight the tight co-localization of the each pair of centrosomal factors in a defined centrosome core structure.

To proliferate, *T*. *gondii* tachyzoites build two daughter parasites, internally enclosing an intact nucleus and a full complement of metabolic and invasion organelles. The daughter buds then consume the residual mother cell to become infectious parasites. The centrosome must be duplicated for this process to unfold, indicating a central organizing function (shown as red dots in diagram, Fig. 1A) [3,4,8]. Budding structures emerge close to the centrosome, and the centrosome is oriented to the unique centrocone compartment embedded in the nuclear membrane that is required for chromosome segregation [25,29]. The molecular basis for the complex coordinating functions of the centrosome in these parasites is not well understood. To decipher the structural features of the *T*. *gondii* centrosome, we mined Apicomplexa genome sequences for conserved centrosomal proteins (see Table 1 and S1 Fig. for gene lists) and examined how these factors are expressed during tachyzoite division. One of the key centrosome structural proteins present in *T*. *gondii* is an ortholog of the cartwheel protein Sas-6 [30]; note that this protein is distinct from the recently described TgSas-6L, which is associated with the apical MTOC [31]. Other canonical centrosomal proteins identified in *T*. *gondii* are centriole elongation factor Sas-4 [32,33], centrin-binding protein Sfi1 [34], gamma-tubulin, and a large protein that contains a single Aurora-like kinase domain (Table 1). The *T*. *gondii* Sas-6 ortholog retains the three-domain structure of the human protein known to be responsible for the 9-fold symmetry of the centriolar barrel (S1A Fig.) [35,36]. The organization of the PISA (Present in Sas-6) motif and Sas-6 conserved domain [30,37] relative to the central coiled-coil domain is also preserved. The major difference between these proteins is the extended and unstructured N- and C-tails of TgSas-6 (S1A Fig.). Epitope tagging the TgSas-6 protein in *T*. *gondii* tachyzoites by genetic knock-in (TgSas-6^HA^, C-terminal 3xHA fusion) demonstrated co-localization with centriolar TgCentrin1^myc^ that was also epitope tagged in the endogenous gene locus in this clone (Fig. 1B and see S2 Table for a full list of transgenic strains used in this study). DAPI (4’, 6–diamidino-2-phenylindole) co-staining was included in this analysis in order to follow the distribution of genomic DNA and to localize the nucleus in the parasites studied. This immunofluorescent microscopy analysis (IFA) validates the centrosome assignment of the TgSas-6 ortholog. We identified an ortholog of the centrin-binding protein Sfi1 (Table 1 and Fig. 1C and S1B and S2 Figs.) first discovered in budding yeast and also present in human cells [34,38]. The TgSfi1 ortholog is a highly disordered protein comprised of multiple short domains and a large disordered C-terminus (1,145 residues, http://www.disprot.org) [39]. A total of 31 divergent centrin binding sites were identified that are evenly distributed over two-thirds of the protein length with none in the disordered C-terminal tail (S1B Fig. and S2 Fig.). Consistent with its known role in pairing with centrin, we found endogenously tagged TgSfi1^myc^ closely co-localized with TgCentrin1^HA^ in the tachyzoite centrosome (Fig. 1C). As expected, *T*. *gondii* γ-Tubulin (Table 1) localized to the centrosome and duplicated along with TgCentrin1-associated structures (Fig. 1D).

**Table 1:** Centrosomal proteins conserved in Apicomplexa phylum.

Protein	Function in eukaryotic centrosome	*Homo sapiens*	*Toxoplasma* ME49_#	*Plasmodium* PF3D7_#	*Cryptosporidium*
**Centriolar proteins**					
**Centrin1**	Ca^++^ binding contractile protein	NP_004335*	247230 (e-63)	0107000 (e-62)	cgd3_1270 (e-53)
**SAS-6**	Cartwheel protein	NP_919268	306430 (e-16)	0607600 (e-31)	cgd8_490 (e-13)
**SAS-4**	Centriole elongation	NP_060921	258710 (e-15)	1458500 (e-16)	cgd4_200 (e-14)
**CP110**	Centriole elongation, SAS-4 antagonist	NP_00118591	none	none	none
**CEP135/Bld10**	Scaffold, centrioles cohesion	NP_000004	none	none	none
**CEP120**	Centriole elongation	NP_694955	285210 (e-18)	0504700 (e-13)	cgd4_770 (e-13)
**CEP97**	Inhibition of cilliogenesis	NP_078824	294930 (e-07)	1032800 ** (e-30)	none
**CEP250/c-NAP**	Centrioles cohesion	NP_009117	212880 (e-86)	1036400 (e-46)	none
**CEP76**	Centriole duplication	NP_079175	226610 (e-59)	0603800 (e-13)	cgd5_780 (e-12)
**Sfi1**	Centrin1 binding partner	NP_001007468	274000 (e-13)	0710000 ***	none
**Pericentriolar matrix (PCM) factors**					
**CEP215/Cnn**	PCM accumulation	NP_001011649	none	none	none
**gamma-Tubulin**	Microtubule nucleation	NP_001061	226870 (e-00)	0803700 (e-00)	cgd7_1980 (e-00)
**CEP192/SPD2**	Mitotic spindle assembly	NP_115518	none	none	none
**PCM recruitment factors**					
**Polo-like kinase 1**	Mitotic spindle positioning	NP_005021	none	none	none
**Polo-like kinase 4**	Centriole duplication	NP_055079	none	none	none
**Aurora kinase A**	Spindle pole stabilization, MT formation	NP_940839	203010 (e-47)	0309200 (e-47)	cgd2_3190 (e-61)

* Human Centrin 2

** BLAST against *Toxoplasma* ortholog

*** No protein sequence similarity, but centrin-binding motifs are present.

### *Toxoplasma* Has an Extended Family of CEP250-Like Proteins

**Fig 2:**
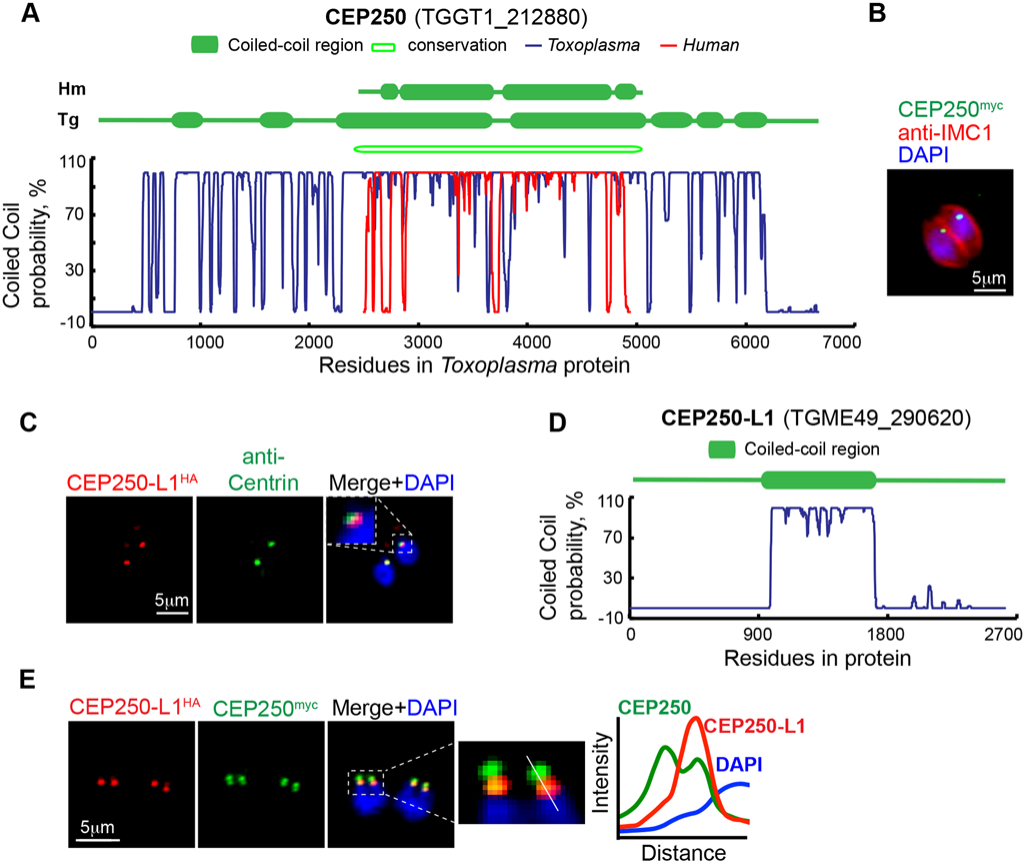
*T*. *gondii* CEP250-related proteins localize to the tachyzoite centrosome. (A) Assessed by primary and secondary protein analysis, TgCEP250 was designated the ortholog of hCEP250. Coiled-coil domains exceeding 200 amino acids are shown in the diagram above the plots of the Marcoil prediction (http://toolkit.tuebingen.mpg.de/marcoil). Variation of the coiled-coil probability for the full length TgCEP250 protein is shown with blue line and for hCEP250 protein with red line. Primary sequence homology was determined by pBLAST and is indicated as an open block in the diagram (conservation). (B) C-terminal epitope-tagging of TgCEP250 (green) protein with 3xmyc by genetic knock-in at the chromosome locus revealed that this factor is localized to a small perinuclear structure consistent with location of the centrosome. Co-staining with DAPI (blue) and anti-IMC1 antibody (red) was used to reference nucleus and the parasite cytoskeleton, respectively. (C) The novel TgCEP250-like protein 1 (TgCEP250-L1) tagged with 3xHA-epitope (genetic knock-in) co-localized near TgCentrin1-positive structures (co-staining with anti-Centrin antibody). Inset panel shows close perinuclear localization of the merged markers. (D) The TgCEP250-L1 protein has a single predicted coiled-coil domain (http://toolkit.tuebingen.mpg.de/marcoil). (E) Co-staining of TgCEP250^myc^ (green) and TgCEP250L-1^HA^ (red) proteins in a transgenic clone, in which the factors were sequentially epitope-tagged by genetic knock-in, shows TgCEP250^myc^ stains four foci in mitotic parasites, while TgCEP250-L1^HA^ co-localized only in the two foci closest to the nucleus. Quantitative fluorescence density plot was built using data taken from a cross section of the image indicated by the white line in the enlarged merged image immediately to the left of the graph.

Studies of the centrosome in higher eukaryotes identified a number of large coiled-coil proteins with important functions associated with centrosome and are, therefore, named the CEP (CEntrosomal Proteins) family of proteins (for review see [40]). Using a combination of BLAST and protein structural analysis, we found several *T*. *gondii* genes encoding proteins similar to the CEP protein family of higher eukaryotes. Apicomplexan orthologs for CEP76, CEP97, CEP120, and CEP250 are listed in Table 1 (structural features shown in S1D Fig. and Fig. 2A). The CEP250/c-Nap1 factor is known to be required for centrosome separation in animal cells [41], and it was of particular interest to determine if this factor was present in tachyzoite centrosomes. The *T*. *gondii* gene encoding a protein with highest similarity to human CEP250/C-Nap is TGME49_212880 (Table 1 and S1 Table). Human CEP250 has coiled-coil domains running across the entirety of the protein length (Fig. 2A, indicated by the red plot line). The *T*. *gondii* TGME49_212880 protein also has numerous coiled-coil domains and appears to preserve an overall architecture of the two central extended helical domains surrounded by short coiled-coil regions (Fig. 2A, blue line and conservation block) as well as having long extended N- and C-terminal tails. To verify centrosomal localization, we tagged TGME49_212880 by genetic knock-in with a 3xmyc epitope. TGME49_212880^myc^ was associated with a discrete perinuclear structure consistent with the position of the centrosome (Fig. 2B). We have designated TGME49_212880 (TgCEP250) as the ortholog of hCEP250.

Curiously, the *T*. *gondii* genome also encodes an abundant complement of coiled-coil factors with significant homology to human CEP250. The ten CEP250-related *T*. *gondii* proteins with highest similarity scores predicted to have extended coiled-coil domains (S1 Table) comprise a group of novel proteins with variable length (from 1,232 to 6,668 amino acid residues). Two of the TgCEP250-like factors on this list were recently described as proteins containing the CRMP domain, were localized to the apical cone (TGME49_244470) or conoid (TGME49_252880) [42], and were not studied here. Two other CEP250-like proteins were endogenously tagged, and immunofluorescence microscopy analysis revealed differential subcellular localizations. Protein encoded by TGME49_242790 gene localized to the peripheral annuli of the parasite cytoskeleton (S3A Fig.), a novel compartment that was previously shown to house TgCentrin2 [43], and therefore, was given the name TgPAP1 (Peripheral Annuli Protein 1). A non-periodic CEP250-related protein encoded by gene TGME49_265840 formed a peculiar fibrous net enclosing nucleus (S3B Fig.) and was named TgNMP1 (Nuclear Mesh Protein 1).

The only other factor in the group of CEP250-like proteins that showed centrosomal localization similar to TgCEP250 was a novel member of the family encoded by the TGME49_290620 gene (Fig. 2C, co-localization with TgCentrin1). The distinguishing structural feature of this CEP250-related factor was a single coiled-coil domain that spanned a central 800 residues within the large 2663 amino acid protein (Fig. 2D). We designated this protein TgCEP250-L1 (TgCEP250-like protein 1). To determine co-localization of this novel centrosomal protein with the related factor TgCEP250, we created a dual-tagged transgenic strain expressing TgCEP250-L1^HA^ and TgCEP250^myc^ (S2 Table). Surprisingly, in tachyzoites undergoing mitosis we observed four TgCEP250-positive perinuclear foci, while TgCEP250-L1^HA^ protein co-localized with only two of the TgCEP250^myc^ positive foci that were always proximal to the nucleus (Fig. 2E; TgCEP250-L1^HA^, red; TgCEP250^myc^, green).

### *Toxoplasma* Centrosomes Have a Unique Bipartite Structural Organization

**Fig 3:**
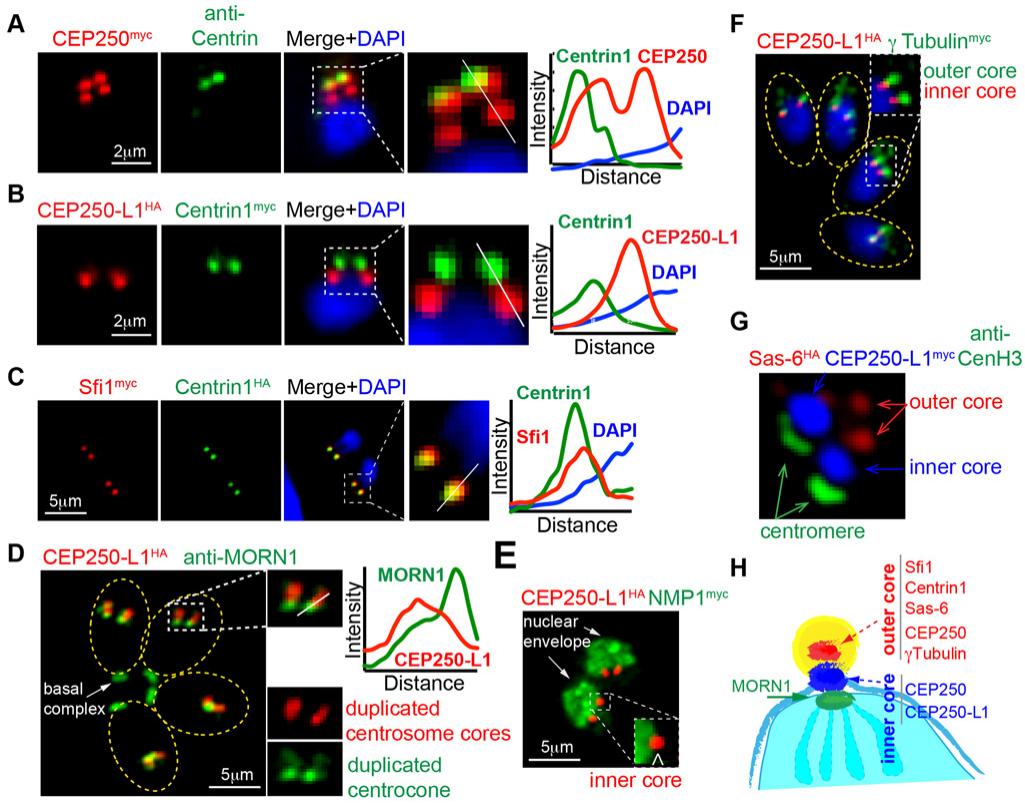
The tachyzoite centrosome has a unique bipartite internal organization. The pairwise localization of several centrosome proteins was examined in tachyzoites undergoing mitosis. Detection of each protein pair was accomplished by epitope-tagging (genetic knock-in) or by using antibody raised against recombinant protein. These experiments revealed two distinct protein core complexes are contained within the tachyzoite centrosome that duplicate during the cell cycle. Two pairwise protein IFA analyses highlight these structures; the (A) upper panel is co-staining of epitope-tagged TgCEP250^myc^ with anti-myc (red) and anti-Centrin (green). Note that TgCEP250^myc^ is localized to the TgCentrin1 core structure (designated the outer core) as well as a novel second core (inner core) that was closer to the parasite nucleus in all cell cycle phases. The (B) panel is a dual epitope-tagged strain TgCEP250-L1^HA^/TgCentrin1^myc^ co-stained with anti-HA (red) and anti-myc (green). The CEP250-related protein, TgCEP250L-1 is preferentially localized to the novel inner core that lacks TgCentrin1. (C) A recognized co-factor of TgCentrin1, TgSfi1 (red), exclusively co-localized with TgCentrin1 (green) in the outer centrosomal core (co-staining of the dual-tagged clone TgCentrin1^HA^/TgSfi1^myc^). Differential composition of the outer versus inner centrosome core also included outer core proteins TgSas-6 and Aurora-related kinase 1 (TgARK1) described below. (D) Duplicated centrosomal cores containing TgCEP250-L1^HA^ were segregated from the centrocone structure embedded in the nuclear envelope as demonstrated by co-staining with anti-TgMORN1 antibody (green). (E) The separation of the inner core from the nuclear envelope was further established by co-staining for the nuclear mesh protein, TgNMP1; analysis of dual-tagged clone TgCEP250-L1^HA^/TgNMP1^myc^. Note that inner core containing TgCEP250-L1^HA^ is localized to the cytoplasmic interface of the nucleus in the inset image. (F) *T*. *gondii* γ-Tubulin is localized to the TgCentrin1-associated outer core (see Fig. 1D) that is distal from the inner core co-stained for TgCEP250-L1^HA^; shown here is dual-tagged clone TgCEP250-L1^HA^/γ-Tubulin^myc^. (G) The spatial segregation of the bipartite core structures of the *Toxoplasma* centrosome is well resolved and aligned with the centromeres as captured by super-resolution microscopy; markers used were outer core = TgSas-6^HA^, inner core = TgCEP250-L1^myc^, and the anti-TgCenH3 antibody to detect the centromere/kinetochore intranuclear structure [25]. For image panels A-D, quantitative fluorescence density plots were included. The data for each density graph was taken from a cross section of the image indicated by the white line in the enlarged merged image immediately to the left of the graph. (H) Diagram is included that summarizes the discovery of two replicating centrosome cores (one-half of the mitotic organization) with differential protein composition.

The discovery of multiple structures in the perinuclear region occupied by centrosomal factors TgCEP250 and TgCEP250L-1 indicated the centrosome of tachyzoites has a complex organization. To investigate this further, we produced several single or dual epitope-tagged transgenic strains (see S2 Table) and in combination with antibodies to TgCentrin1, TgMORN1 (centrocone), or TgCenH3 (centromere/inner kinetochore) examined in detail the structural organization of the parasite centrosome and associated mitotic nuclear structures. An unexpected result of these studies was finding that the TgCentrin1 marker commonly used to identify centrosomes in these parasites was contained in only one of two internal protein core structures of the tachyzoite centrosome. The TgCentrin1-associated protein core was named the outer core because it was always distal to the tachyzoite nucleus (Fig. 3A), while an inner core (protein complex with immediate perinuclear orientation) lacking TgCentrin1 harbored proteins TgCEP250^myc^ and TgCEP250-L1^HA^ (Fig. 3A and B, and Fig. 2E). Paired with TgCentrin1^HA^, the TgSfi1^myc^ protein was exclusively localized to the outer core (Fig. 3C) as was the centriole cartwheel protein TgSas-6 (dual tagged strain, TgSas-6^HA^/TgCentrin1^myc^, Fig. 1B). The TgCEP250-L1^HA^-associated inner core structure was closely aligned, but resolved from the centrocone in late mitosis (Fig. 3D, anti-TgMORN1 co-staining) [29]. Co-staining with putative nuclear envelope factor TgNMP1^myc^ placed the inner core on the outside of the nucleus (Fig. 3E, TgCEP250-L1^HA^/TgNMP^myc^). TgCEP250^myc^ protein was associated with both types of core structures in mitotic tachyzoites (Fig. 3A), while TgCEP250-L1^HA^ showed more exclusive association to the inner core (Fig. 3B) with transient translocation to the outer core providing possible linkage between two structures (Fig. 3D). *T*. *gondii* γ-Tubulin^myc^ showed preferential localization to the TgCentrin1-containing outer core and not to the inner core (Fig. 3F, CEP250-L1^HA^/γ-Tubulin^myc^). Finally, the visualization of the two cores (TgSas-6^HA^ and TgCEP250-L1^myc^) with the centromere/inner kinetochore complex (TgCenH3) by structured illumination microscopy (Fig. 3G, Sas-6^HA^/CEP250-L1^myc^/anti-CenH3) confirmed that the parasite centrosome contained two novel cores that aligned to the kinetochore constituting a multi-layered mitotic machinery spanning the nuclear membrane of the dividing tachyzoite. A diagram (Fig. 3H) summarizes the protein composition and alignment of the two centrosomal cores in one-half of a dividing nucleus.

**Fig 4:**
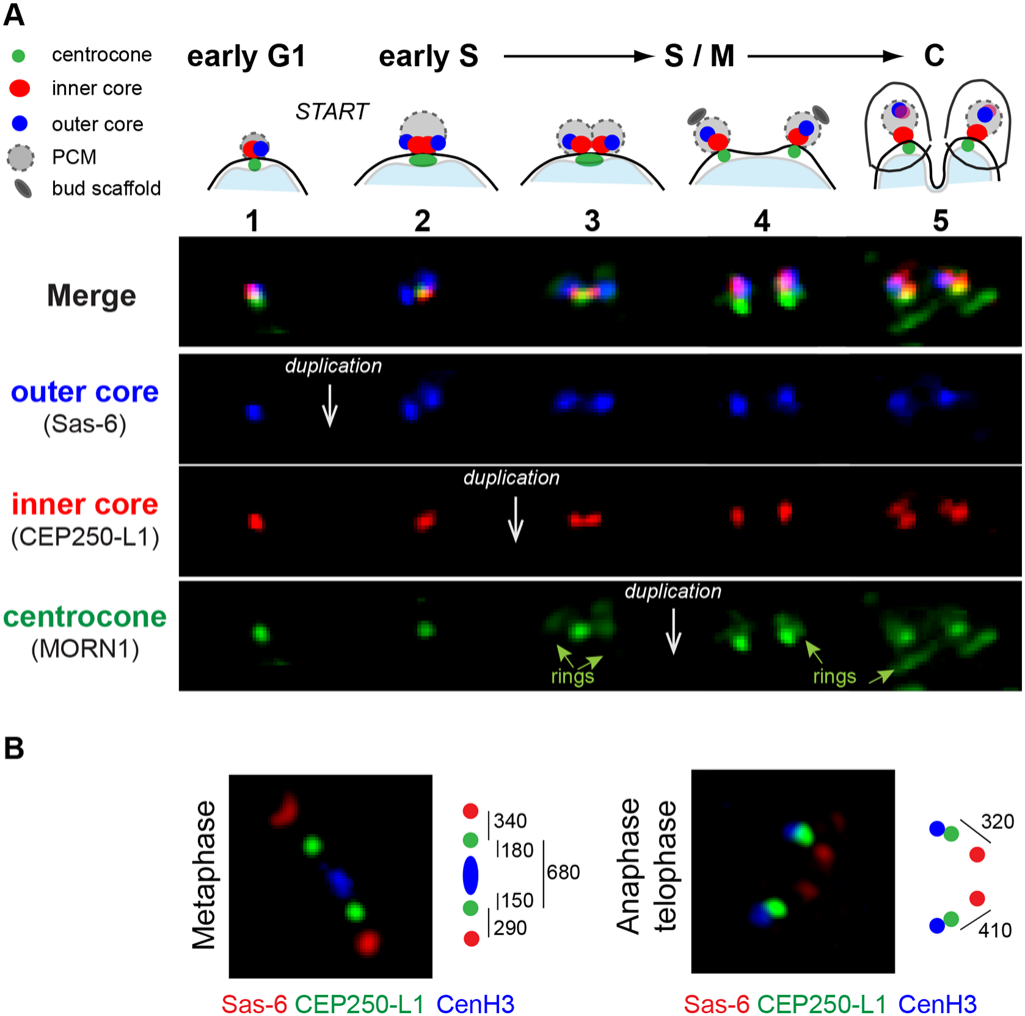
Morphogenesis of the centrosome during the tachyzoite cell cycle. (A) Morphological transitions of the outer and inner protein core complexes were monitored in the dual epitope-tagged TgSas-6^HA^/TgCEP250-L1^myc^ clone. Co-staining with antibodies against TgMORN1 was used to visualize the nuclear membrane centrocone compartment (spindle pole). Above the fluorescent merged images (Merge) a diagram of the composite structures is placed within the cell cycle timing of the biosynthetic events. Five distinctive morphological transitions were identified, and three sequential duplication events (white arrows) were recorded in the following order: duplication of the outer core (TgSas-6^HA^, blue panel) followed by the inner core (TgCEP250-L1^myc^, red panel) and lastly, the centrocone (anti-TgMORN1 antibody, green panel). The cell cycle stages for each transition of the centrosome cycle were determined using well-established nuclear and cellular morphologies. (B) Super resolution images of the centrosome cores (TgSas-6^HA^/TgCEP250-L1^myc^) in relation to the centromeres (visualized with anti-TgCenH3 antibody) in the tachyzoite metaphase and anaphase/telophase. The diagram to the right of each image estimates the physical position and distance between the middle points of the three mitotic structures: outer and inner centrosome cores and kinetochore.

To understand the duplication of the unusual tachyzoite centrosome, we followed the development of the dual centrosome cores and the centrocone in all phases of the parasite cell cycle (Fig. 4A: TgSas-6^HA^, blue; TgCEP250-L1^myc^, red; anti-TgMORN1, green). Newly formed G1 parasites inherit a single, condensed centrosome as demonstrated in the first series of images (Fig. 4A, vertical series 1). The compact G1-centrosome was tightly associated with the nuclear envelope and enlarged during G1 progression. In late G1, the outer core containing TgSas-6^HA^ expanded and duplicated (Fig. 4A, series 2). Simultaneously, the inner core containing TgCEP250-L1^myc^ separated from the outer core, although it retained close association with the nuclear centrocone (Fig. 4A, series 2 green stain). The expanded inner core duplicated immediately after the outer core had replicated, also retaining the distinctive spatial orientation with respect to the nucleus, inner core proximal, and the outer core distal (Fig. 4A, series 3). The duplication of the cores prepared the tachyzoite for mitosis and analysis by super-resolution microscopy demonstrated that the cores aligned with the TgCenH3 containing inner kinetochore [25] in a typical metaphase relationship (Fig. 4B). The two outer cores (TgSas-6^HA^, red stain), two inner cores (TgCEP250-L1^myc^, green stain), and centromeres (anti-TgCenH3, blue stain) were aligned in a linear array extended 1,300 nm from one outer core to the other. At this mitotic stage the distance between each pair of outer and inner centrosome cores measured 300 nm, reaching a maximum of 400 nm in anaphase and telophase (Fig. 4B). The nuclear centrocone containing TgMORN1 protein split in two last in the observed mitotic sequence (Fig. 4A, series 4). The distinct centrosome core structures segregate into each daughter parasite (Fig. 4A, series 5) indicating there is a physical mechanism that ensures each new parasite receives one copy of each type of centrosome core.

### Distinct Protein Core Complexes in the Tachyzoite Centrosome Are Independently Controlled

**Fig 5:**
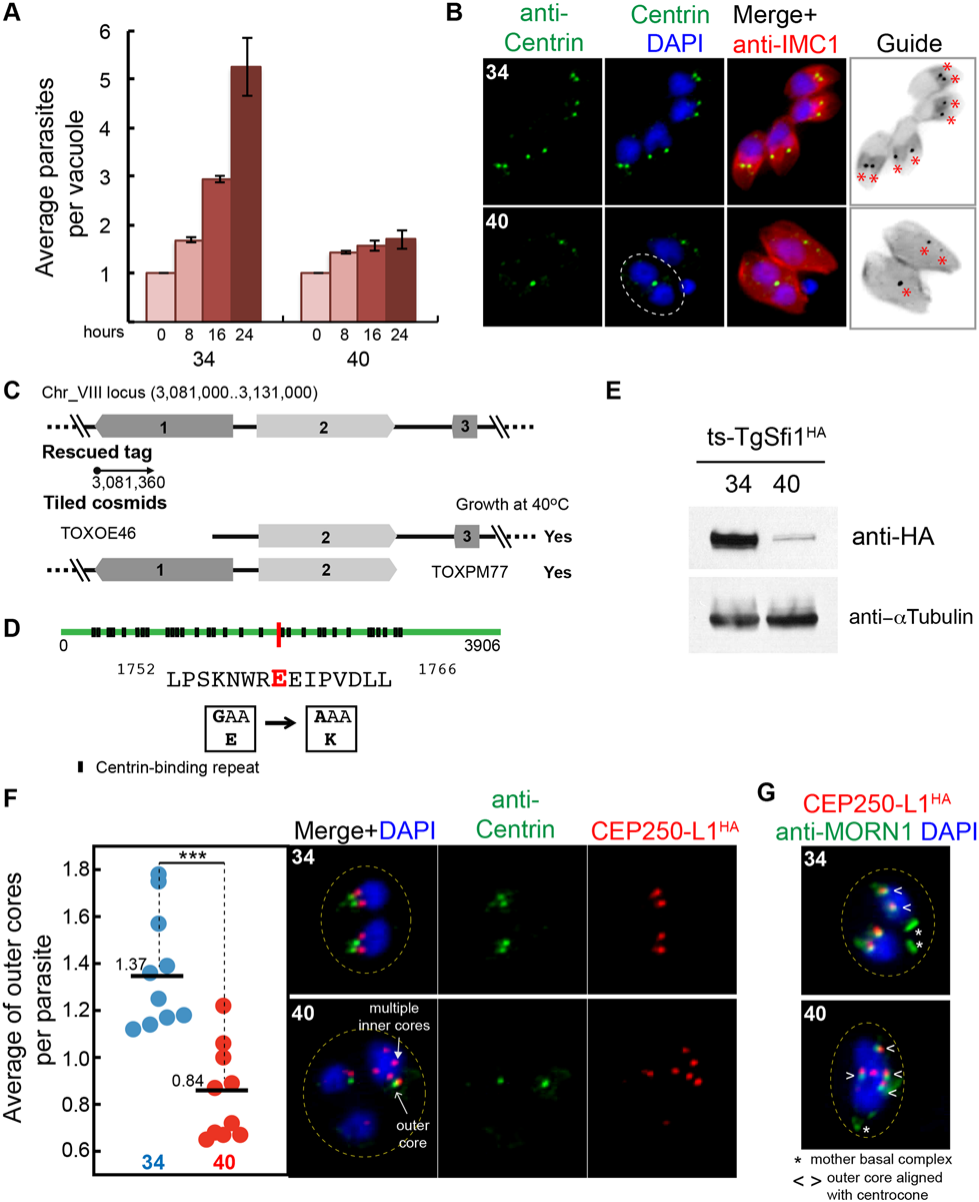
The outer centrosome core protein TgSfi1 has an essential role in the parasite cell cycle. (A) Growth of the chemical mutant 9–86E4 is inhibited by high temperature, leading to lethal arrest (40°C). Cultures were pre-synchronized by limited invasion, and the average number of parasites per vacuole was calculated after 0, 8, 16, and 24 h at indicated temperature. Bar graph shows mean values and standard deviations for three growth experiments with a minimum of 50 vacuoles per time point (for all raw data, see S1 Data). (B) Duplication of the outer core detected by anti-Centrin staining is severely affected in the 9–86E4 mutant grown at 40°C for 20 hours but not when the mutant is grown at the permissive temperature (34°C). Culture temperatures are indicated in the upper left of each image panel. The included guide panel is a non-colored inverted image of the merged red (anti-IMC1) and green (anti-Centrin) stains. Red asterisks indicate the position of the Centrin-positive structures. Note the parasite at 40°C with duplicated nuclei with a single TgCentrin1-associated core (circled) where, normally, there should be two cores. (C) Genetic complementation of mutant 9–86E4 with cosmid genomic libraries identified the defective locus on chromosome VIII (see top diagram) and this was further resolved to gene 2 (TGME49_274000) with individual cosmids that span the locus; gene 2 encodes centrin co-factor, TgSfi1 (see S1B Fig. and S2 Fig.) [38]. (D) Whole genome sequencing of mutant 9–86E4 independently confirmed the ts-TgSfi1 protein was mutated with a E1759K change shown by a red bar in the TgSfi1 protein diagram. Putative centrin binding sites (black box) are also indicated (see also S2 Fig.). (E) Western blot analysis of the mutant 9–86E4 parasites expressing ts-TgSfi1^HA^ protein after 24 h growth at 34°C or 40°C. Total lysate of 20 x 10^6^ parasites were probed with anti-HA-epitope or anti-α-Tubulin antibodies. (F) The average of TgCentrin1 containing outer cores (anti-Centrin staining) per parasite, determined by anti-IMC1 staining of the mother parasite, was quantified in ten microscopic fields with an average of 3–10 vacuoles (for all raw data, see S1 Data) revealing a significant reduction of the outer cores when mutant 9–86E4 was shifted to 40°C (red dots). *P*-value ≤ 0.0005 (***) was calculated using paired two-tail *t* test. The loss of the outer core at high temperature in the ts-TgSfi1 mutant does not prevent replication of the inner core. To monitor inner core, TgCEP250-L1^HA^ was introduced in the mutant 9–86E4 (see Generation of transgenic tachyzoite strains in Materials and Methods). Co-staining of TgCEP250-L1^HA^ (inner core) and anti-Centrin (outer core) showed that amplification of the inner core occurred in parasites in which duplication of the outer core was inhibited. (G) Multiple inner cores (TgCEP250-L1^HA^, red) in mutant 9–86E4 parasites at 40°C showed tight alignment and matched duplication of the nuclear centrocone (anti-TgMORN1stain, green).

The discovery of two distinct centrosome core complexes and the association of TgCentrin1 to only one core [44] indicates the *Toxoplasma* centrosome is more complex than previously appreciated. We turned to the large collection of conditional cell cycle mutants generated by earlier studies [21,27] for insight into their interdependence and regulation. Among the group of mutants with defects in mitosis, the mutant 9–86E4 possessed a temperature-sensitive allele of the outer core protein TgSfi1 (see characterization of the protein in Figs. 1C, 3C, S1B, S1C, and S2). Mutant 9–86E4 parasites grew normally at the permissive temperature of 34°C, while at 40°C they quickly growth arrested (Fig. 5A). At 40°C mutant 9–86E4 parasites were typically larger than parasites grown at 34°C, although this was not systematically quantified. At high temperature, there was absence of budding. Parasites were able to duplicate the nucleus (see circled parasite in Fig. 5B, 40°C panel), but with the primary budding defect this led to unequal DNA and nuclei distribution with rare DNA-free zoites present. Genetic complementation, followed by marker rescue (Fig. 5C) [23,26] and whole genome sequencing of mutant 9–86E4 (see “Identification of Temperature-Sensitive Mutations” in Materials and Methods) [45,46], pinpointed a E1759K mutation in the TgSfi1 gene as responsible for these growth defects (Fig. 5D). To monitor the ts-allele of TgSfi1 protein, we introduced 3xHA-epitope into the ts-TgSfi1 locus using modified CRISPR technology [47]. Western blot analysis of the mutant parasites expressing ts-TgSfi1^HA^ showed high instability of the tagged protein after 24 h incubation at 40°C (Fig. 5E), indicating that parasites grown at 40°C were phenotypically null for this factor. Consistent with a centrosomal function, and as a known partner with Centrin [48], the loss of ts-TgSfi1 led to dramatic reduction in the TgCentrin1-associated cores in the 9–86E4 mutant at 40°C (Fig. 5B and F).

To determine how the inner core containing TgCEP250-L1 was affected by the loss of ts-TgSfi1 at high temperature, we introduced into the 9–86E4 mutant a C-terminal tagged version of the TgCEP250-L1 protein regulated by its own promoter (see fosmid construction in “Generation of Transgenic Tachyzoite Strains” of Materials and Methods). The resulting transgenic strain carrying the ts-TgSfi1(E1759K) mutation was used to visualize both the outer (anti-Centrin, green) and inner core (TgCEP250-L1^HA^, red). At the permissive temperature, parasites maintained the expected 1:1 ratio of outer/inner cores (Fig. 5F, average 1.37 at 34°C in a population average from an asynchronous culture comprised of 60% G1 and 40% S and M/C parasites). However, a shift to 40°C reduced the number of the TgCentrin1-outer cores below the minimal one per nucleus (Fig. 5F, dot plot: 0.8 at 40°C versus 1.4 at 34°C), while the number of TgCEP250-L1^HA^-inner cores dramatically increased beyond the proper 1:1 nuclear stoichiometry (Fig. 5F, red). While the TgCentrin1 outer cores were reduced, the few outer cores that remained were maintained in the normal distal perinuclear position observed in normal replicating parasites. By contrast, over-amplified inner TgCEP250-L1 cores decorated the nucleus in a pattern that was independent of the remaining outer TgCentrin1 cores, indicating the position and replication of the inner core structures was uncoupled from the outer core. Intriguingly, the amplification of inner TgCEP250-L1 cores was always paired with and orientated to replicated TgMORN1-associated centrocone structures, suggesting a common mechanism was directing the amplification of both structures (Fig. 5G). Therefore, the loss of ts-TgSfi1 limited function of the outer core at the restricted temperature, which caused blocking of the outer core duplication while loosening control of the single duplication of the inner core. The other consequences of the defect were physical separation of two cores and severe restriction of daughter budding.

### A MAPK-Like Protein Kinase Is Required for Proper Daughter Budding and Mitosis

**Fig 6:**
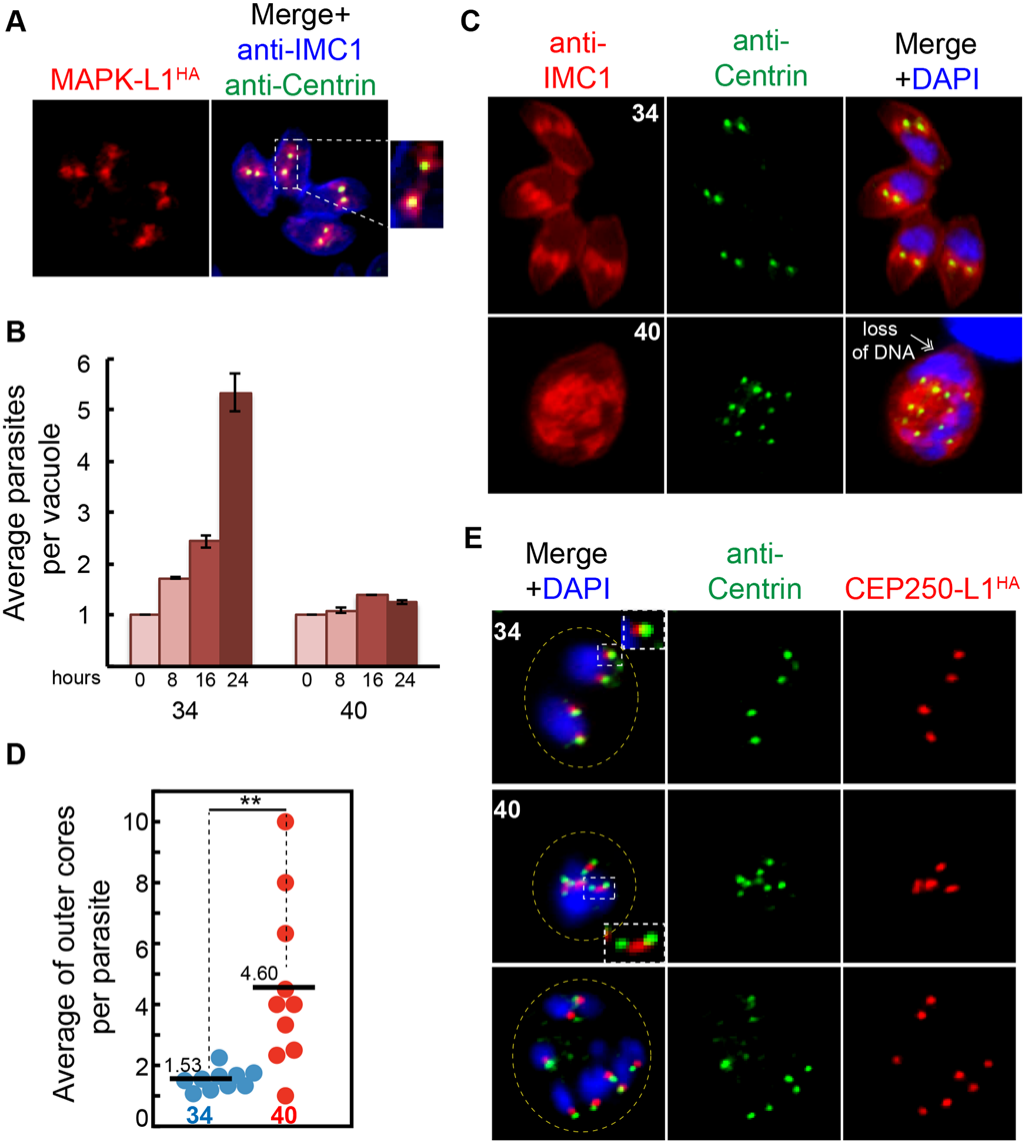
TgMAPK-L1 localizes to the centrosome and controls proper duplication. (A) Co-staining of the endogenously tagged TgMAPK-L1^HA^ with anti-Centrin and anti-IMC1 antibodies. Magnified image on the right shows a vacuole of four parasites post-duplication of the centrosome surrounded by TgMAPK-L1^HA^ in the pericentrosomal matrix (PCM). (B) Mutant 11–31G12 parasites defective in TgMAPK-L1 (see details in S4 Fig.) quickly growth arrested at 40°C. Growth of the mutant at 34°C and 40°C was quantified as described in the Fig. 5A (for all raw data, see S1 Data). (C) Disruption of ts-TgMAPK-L1 function resulted in the over-duplication of the outer centrosomal cores (anti-Centrin, green) accompanied by defects in chromosome segregation and nuclear division (double-headed arrow). (D) Quantification of the Centrin-containing outer cores per original mother parasite (determined by anti-IMC1 staining) in mutant 11–31G12 parasites grown at 34°C (blue dots) and 40°C (red dots) for 20 h is shown in the dot plot (for all raw data see S1 Data). Mean values for each dataset are also indicated (black bars). Significant difference between two conditions was verified by paired *t* test that returned the *p*-value ≤ 0.007 (**). Note that while both cores are amplified beyond the normal stoichiometry at 40°C, there is not a precise match in the total number of outer and inner cores in every parasite. (E) To determine the fate of the inner centrosomal core, the TgCEP250-L1 protein epitope-tagged with 3xHA (red) was introduced into mutant 11–31G12 parasites, producing a transgenic clone, 11–31G12(TgCEP250L-1^HA^). Co-staining of this clone with anti-HA (red) and anti-Centrin antibodies (green) showed that at 40°C the inner cores were multiplied along with the outer centrosome cores while preserving proper spatial alignment. Duplication of the inner core was slightly delayed, leading to accumulation of the intermediate “dumbbell” structures shown in the inset of the middle 40°C merged panel. Yellow dotted line indicates vacuole boundary in the infected host cell shown in these images.

The uncoupling of the replication of outer and inner cores in the ts-TgSfi1 mutant above indicates that complex regulatory mechanisms operate in the *Toxoplasma* centrosome to control duplication of the centrosome cores. To investigate the replication of these cores further, we studied a serine/threonine protein kinase related to mammalian ERK1, which previous studies determined is required for tachyzoite growth, although the underlying mechanism responsible was not reported [45]. The gene TgME49_312570 is one of three protein kinase genes in *T*. *gondii* possessing a MAPK-like kinase domain [49]. The similarity of TGME49_312570 to eukaryotic MAPK factors lies almost exclusively in the ATP binding pocket, which corresponds to approximately 270 amino acids (aa) of an otherwise 1,298 aa novel protein. Given the lack of the MEKK-MEK-MAPK signal transduction module in the Apicomplexa [50], a similarity limited to part of the kinase domain in TGME49_312570 and no established mechanistic function for this protein, we have designated this gene as TgMAPK-like 1 (TgMAPK-L1). Epitope tagging of this protein by genetic knock-in demonstrated a prominent pericentrosomal pattern surrounding the TgCentrin1 outer core in the tachyzoite S-phase and early in mitosis (TgMAPK-L1^HA^; Fig. 6A and S4A Fig.). During budding, expression of the TgMAPK-L1^HA^ rapidly decreased and dropped below detection level in the newly emerged G1 parasites, consistent with the cyclical pattern of the encoded mRNA (protein cell cycle properties; S4A Fig.; for mRNA profile see Toxodb). Similar staining is often seen for proteins localized in the pericentriolar matrix (PCM) [51], which, because of low conservation of PCM markers, have not been identified in *T*. *gondii*. The pericentrosomal localization of TgMAPK-L1 makes this protein a first candidate for the PCM compartment.

There are few reports in animal cells of MAPKs exclusively localized to the centrosome as we observed for TgMAPK-L1^HA^ in tachyzoites, nor is direct mitotic control the mechanism typically associated with MAPKs of higher eukaryotes, in which signal transduction in response to external growth factors is the more common function [52]. Insight into the function of TgMAPK-L1 was provided by a temperature-sensitive mutant, 11–31G12, recently identified in the large collection of tachyzoite cell cycle mutants [21]. Mutant 11–31G12 parasites carrying a L534Q mutation immediately C-terminal of the TgMAPK-L1 kinase domain (S4 Fig.) rapidly growth arrest at 40°C (Fig. 6B) with defects in the coordination of daughter budding with mitosis leading to abnormal numbers of internal daughters and nuclei (Fig. 6C, 40°C panel). The unlinking of parasite budding and nuclear duplication in the mutant led to an elevated ratio of centrosome to bud numbers, as evident from anti-Centrin staining (Fig. 6D, dot plot: 1.53 at 34°C versus 4.6 at 40°C). Genetic complementation followed by marker rescue and ts-allele sequencing identified ts-TgMAPK-L1 as responsible for the high temperature defects (S4B Fig. and S4C Fig.). To verify that the mutation in ts-TgMAPK-L1 was solely responsible for the cell cycle defects, we transferred the L534Q mutation into parent RHΔ*ku80* parasites and simultaneously tagged the ts-TgMAPK-L1 protein with three copies of the HA epitope (S4D Fig.). The introduction of the L534Q mutation recapitulated the temperature and cell cycle defects (S4E Fig.) of the 11–31G12 mutant. After 20 h at 40°C, tachyzoites carrying the ts-TgMAPK-L1 mutation were yet to produce a mature daughter parasite with multiple buds forming in a single mother cell (S4E Fig., anti-IMC1 panel), indicating there is a loss of the critical controls that restrict binary division in these parasites. Western blot and IFA analysis of the ts-TgMAPK-L1^HA^ protein (S4E,F Fig.) indicated that instability of this protein at high temperature creating a null phenotype is the major cause of conditional growth arrest in ts-TgMAPK-L1 mutant parasites.

**Fig 7:**
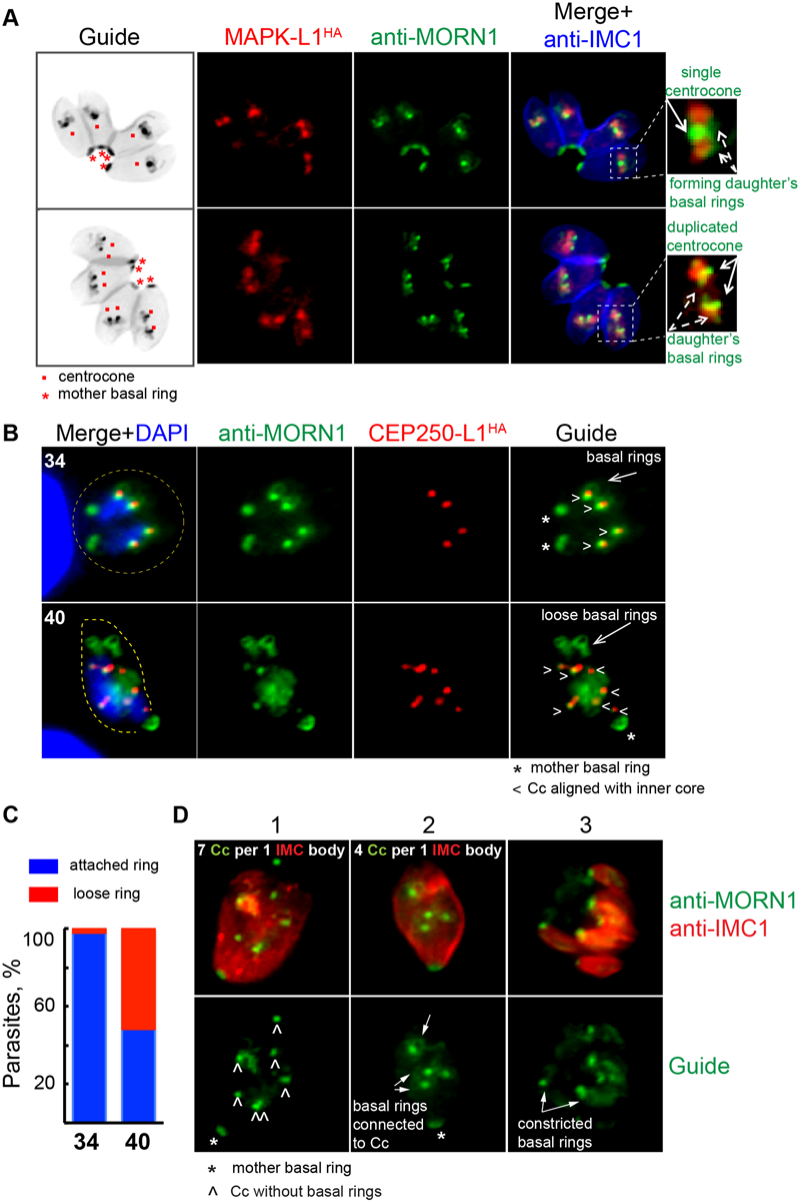
TgMAPK-L1 has a key role in coupling mitosis and cytokinesis. (A) In replicating parasites endogenously tagged wild-type TgMAPK-L1^HA^ (red stain) showed a predominant alignment with single (upper panel) and duplicated (lower panel) centrocone (Cc; anti-MORN1 staining), surrounded by the TgMORN1-rings (new daughter’s basal complexes). Non-color guide panels of the inverted merged images of the anti-IMC1 (blue channel) and the anti-MORN1 (green channel) staining were included here to provide a reference to the location and scale of the subcellular structures observed within the parasite. Magnified merged images to the right of each panel are included to provide greater detail of the key structures stained. (B) Amplified inner cores (TgCEP250-L1^HA^) in ts-TgMAPK-L1 mutant 11–31G12 parasites arrested at 40°C for 20 h maintained alignment with the nuclear centrocone (anti-TgMORN1 staining); however, MORN1-rings (daughter’s basal rings) were no longer attached to duplicated centrocones in many parasites. Asterisks indicate position of the basal complex of the mother cell and “<” points to the aligned inner core/centrocone structures. Examples of disconnected basal rings are evident in the lower 40°C panel (long arrow), while at the permissive temperature (34°C) the daughter’s basal rings are tightly connected to the centrocone. Yellow dashed line in the merged images indicates the parasite size (40) or the size of the vacuole (34). (C) Bar graph shows quantification of the disruption of the daughter’s MORN1-ring attachment to the centrocone. Only S/M stages that normally display MORN1-rings were quantified at 34°C. Vacuoles with detectable MORN1-rings were analyzed at 40°C. No less than 100 vacuoles were analyzed for each condition (for all raw data see S1 Data). (D) Co-staining of the nuclear centrocone and basal rings (anti-TgMORN1 antibody, green) with the membrane scaffold (anti-IMC1 antibody) in the ts-TgMAPK-L1 parasites arrested at 40°C for 20 h. Merged images are shown in the upper panel. Anti-TgMORN1 stain in the Guide panel highlights only the centrocone and basal ring relationships. The three images are representative of the predominant morphological defects associated with ts-TgMAPK-L1 in mutant parasites. First image represents an early step in the development of phenotype: mother cell with multiplied centrocones (7) and no visible formation of the daughter’s basal rings (“^”). The mother cell in the second image similarly has amplified centrocones (4), however, they are connected to the well-developed daughter MORN-rings (arrows). The consecutive step of abnormal multiple budding (7) is represented on the third image. Note that each daughter cell has a constricted basal ring (arrow) at the proximal end of the body.

To further investigate the mechanism of irreversible and lethal growth arrest caused by ts-TgMAPK-L1, we introduced 3xHA-tagged TgCEP250-L1 into the original ts-TgMAPK-L1 mutant, 11–31G12, and with the use of anti-Centrin antibody analyzed the outer and inner centrosome cores in this mutant. Upon shift to high temperature, we observed rapid amplification of both centrosomal cores that roughly maintained the internal alignment (Fig. 6E; anti-Centrin, green; TgCEP250-L1^HA^, red). Replication of the inner core was often delayed in the mutant, leading to accumulation of the distinctive “dumbbell” forms (Fig. 6E, inset 40°C panel). We next examined the relationship between the centrosome and developing daughter structures and how the loss of ts-TgMAPK-L1 in the centrosome affected critical features of daughter budding in mutant parasites (Fig. 7). In these experiments we monitored TgMORN1, which is present in the distinctive spindle-associated nuclear centrocone and early (daughter) basal ring [29]. Both of the basal ring and centrocone structures are tightly associated in a single complex in the parasite S phase and early mitosis (Fig. 7A and 7B, 34°C) and are in close proximity to the PCM localized TgMAPK-L1^HA^ in wild-type tachyzoites (Fig. 7A, magnified merge images on the right). Although deficiency in ts-TgMAPK-L1 in mutant parasites did not affect alignment of the nuclear centrocone with centrosome (Fig. 7B; 40°C; lower panel; anti-TgMORN1, green; inner core visualized with TgCEP250-L1^HA^, red), it severed stable associations between the centrocone and the early forming daughter basal rings (Fig. 7B, 40°C panel, loose basal rings). Disconnection of the basal ring and centrocone compartments was observed in 50% of the ts-TgMAPK-L1 parasites at high temperature (Fig. 7C). Further analysis of the ts-TgMAPK-L1 mutant revealed that a delay in basal ring development accompanied by subsequent karyokinetic counting defects was the prevalent phenotype of ts-TgMAPK-L1 parasites at 40°C. The images of co-markers TgMORN1 and TgCEP250-L1^HA^ in the Fig. 7D illustrate three consecutive stages of the centrocone/basal ring uncoupling defect in ts-TgMAPK-L1 parasites. Note that under normal conditions, the cell cycle length of *T*. *gondii* tachyzoite used in this study is 8 h at 37°C [7,10], and by 20 h post-infection, replicating parasites typically complete three cell cycles in a single infected host (four to eight parasites per vacuole). By contrast, mutant 11–31G12 parasites at 40°C do not divide following invasion, forming large cells that retain the original mother cell IMC1 and basal complex (Fig. 7D; panels 1 and 2; anti-IMC1, red; anti-TgMORN1, green). The majority of ts-TgMAPK-L1 deficient parasites had over-amplified centrocones, with a distinct subpopulation also failing to form daughter basal rings (Fig. 7D, panel 1). The lack of basal rings appeared to induce another round of nuclear duplication in the absence of budding. In other parasites, we observed the formation of daughter basal rings that retained the proximity to the centrocone (Fig. 7D, panel 2) leading to the assembly of multiple abnormal daughter buds (Fig. 7D, panel 3). At high temperature, all daughter formation in these vacuoles was nonviable due to numerous mitotic defects (Fig. 6C, retention of the mother DNA in the lower panel). Together, these results indicate TgMAPK-L1 has a specific role in restricting tachyzoite nuclear replication that likely involves the preservation of the physical connection between the karyokinetic and cytokinetic centers.

### Novel *Toxoplasma* Aurora Kinase Localizes to the Outer Core of the Centrosome

**Fig 8:**
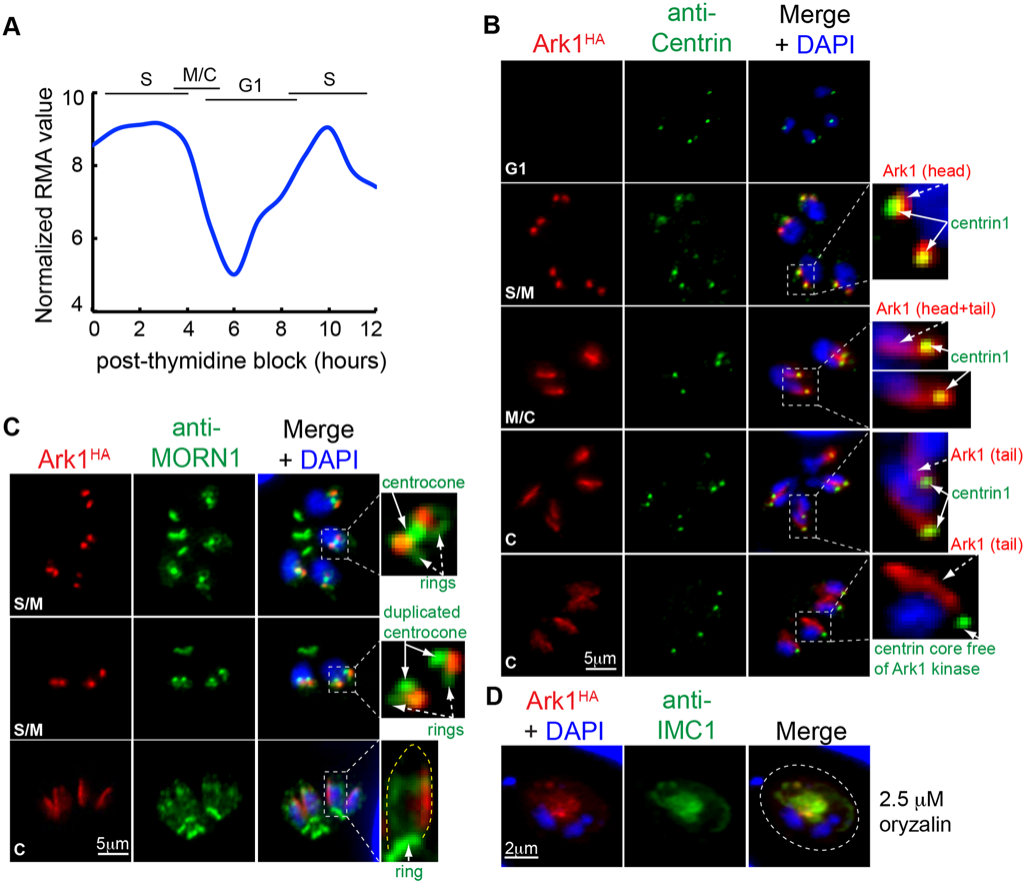
A novel *Toxoplasma* aurora-related kinase shows dynamic association with parasite cytoskeletal structures. Periodicity of TgArk1 mRNA (A) translates into dynamic cell cycle protein expression (B) (for raw mRNA data, see S1 Data). At peak expression in S/M phase, TgArk1^HA^ protein (red, 3xHA tagged in genome by knock-in) was co-localized to the centrosome outer core complex as demonstrated by the anti-HA (red) and anti-Centrin (green) co-staining. Intriguingly, as parasites entered mitosis, TgArk1^HA^ also became localized to a discrete linear structure. The dual pattern of localization in the centrosome outer core (TgArk1, head morphology) and the novel linear structure (TgArk1, tail morphology) was altered further in late cytokinesis (last two panels) when TgArk1 disappeared from the TgCentrin1-associated outer core complex. Magnified images of the specific areas within the merged panels are shown to the right. Cell cycle stages are indicated in the upper left of each image series. (C) Co-staining with anti-TgMORN1 (green) antibody showed that association of TgArk1^HA^ with the centrosome is maintained during centrocone development and duplication. The linear structure stained by TgArk1^HA^ (tail morphology) is unusual and runs along one side of the growing daughter bud. Yellow dotted line in the magnified image of the cytokinesis stage represents a cytoskeleton of the growing daughter bud. Cell cycle stages are indicated in the upper left of each image series. (D) Treatment of tachyzoites with 2.5 μM oryzalin for 24 h to disrupt subpellicular microtubules indicates the linear TgArk1^HA^-tail is an unknown cytoskeletal structure, although the primary association may also involve the factors of the inner membrane complex because TgArk1^HA^ staining was retained with the disrupted IMC1-containing material after oryzalin caused disassembly of the daughter subpellicular microtubules. Dotted line outlines boundary of the parasitophorous vacuole in the infected host cell.

It had been shown that several mitotic kinases, including cyclin-dependent, Polo-like, Aurora, and NIMA-related kinases, coordinate timing and fidelity of the centrosome function in higher eukaryotes [53]. Similarly, a recently identified *T*. *gondii* ortholog of the NIMA-related kinase, TgNek1–2, showed dynamic association with the centrosome in which it positively regulated the TgCentrin1-core complex duplication [54]. Another group of the conserved eukaryotic kinases is Aurora family, which plays a central role in the establishment of the bipolar spindle [55]. A single gene in the *Toxoplasma* genome encodes a protein possessing a kinase domain similar to Aurora kinases of higher eukaryotes [56,57]. The single exon gene (Table 1) encodes a large, 2,812 amino acid protein that is dynamically regulated over the tachyzoite cell cycle at the mRNA and protein level (Fig. 8A and B). We epitope tagged TgAurora-related kinase 1 (TgArk1) with 3xHA and discovered by IFA analysis that this regulatory factor provides another example of the heterologous composition of the centrosome core structures in this parasite. As predicted by the mRNA profile, TgArk1^HA^ was not detected in G1 parasites but quickly reached maximum expression in S phase and gradually disappeared from parasites containing mature daughter buds (Fig. 8B, red). In S phase and early mitosis, the TgArk1^HA^ factor was localized exclusively to the TgCentrin1-associated outer core of the parasite centrosome (Fig. 8B, TgArk1-head morphology) and not in the centrocone compartment that is visualized using anti-TgMORN1 antibody (Fig. 8C) [24,29,58]. Later in mitosis, TgArk1^HA^ became associated with a linear structure (Fig. 8B and C, TgArk1-tail morphology) lying along one side of the growing daughter bud. To determine whether TgArk1-tail associated with nuclear or microtubule structures, we treated TgArk1^HA^ transgenic parasites with microtubule disrupting agent oryzalin. In the absence of the assembled subpellicular microtubules, the TgArk1-tail associated with the inner membrane disrupted material (Fig. 8D, anti-IMC1 co-staining) and not with the parasite nucleus (Fig. 8D, DAPI).

## Discussion

**Fig 9:**
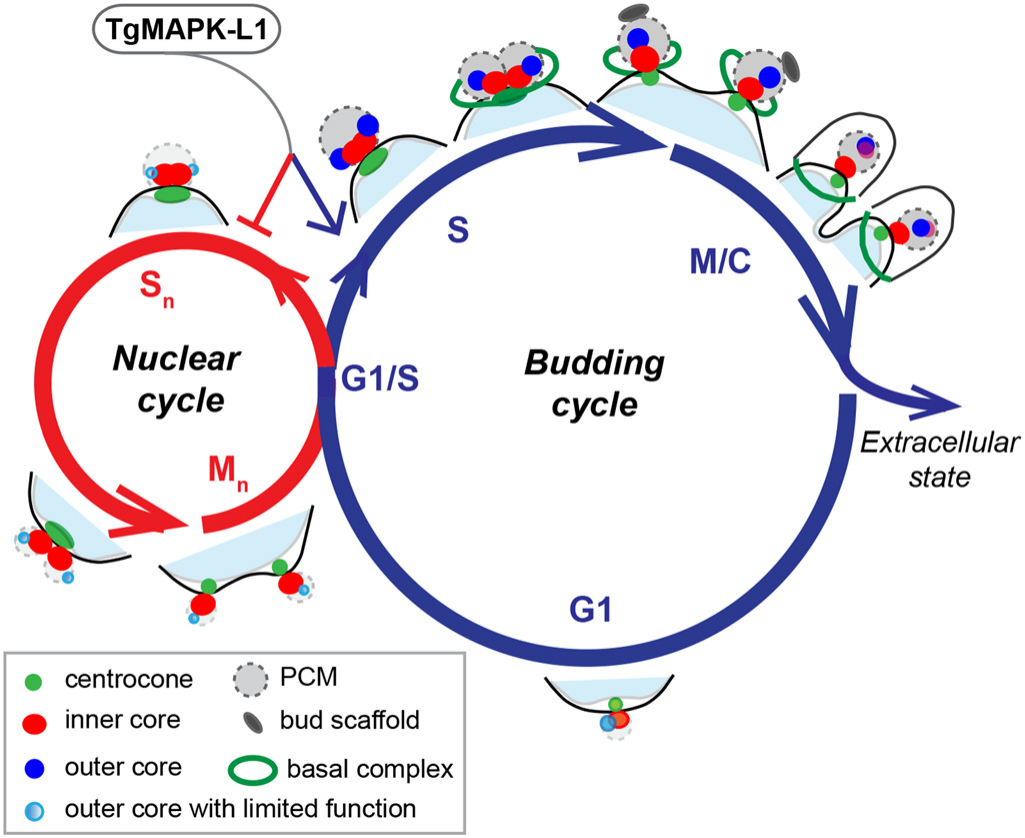
Regulation of the apicomplexan centrosome cycle may be key to cell division flexibility. The two basic cell cycles responsible for Apicomplexa replication are diagrammed with the proposed centrosome cycles overlaid. Entering the single **budding cycle** produces daughter parasites, while multiple **nuclear cycles** amplify chromosome number in the absence of budding. In this model, modulation of the complex centrosome is proposed to regulate the outcome of each cell division cycle. Active mechanisms associated with the outer centrosome core (bright blue circle) control the initiation of budding (green open ring, basal complex; gray oval, bud scaffold), while inactivation of this core (light blue circle) may be required to suspend budding in the nuclear cycle. In the absence of an active outer core, the inner core and centrocone permit each nucleus to complete chromosome replication and mitosis. Our study points to a key PCM factor, TgMAPK-L1, as a regulator of counting in the budding cycle of tachyzoites. Operating at the G1/S boundary, we propose that TgMAPK-L1 may promote and restrict the biosynthetic outcome of the budding cycle (blue arrow), while blocking a path into the nuclear cycle (red arrow).

The *T*. *gondii* tachyzoite stage studied here divides using a budding cycle that is limited to one round of genome duplication (endodyogeny), which is a restriction lifted in the merozoite stage of the cat life cycle that replicates by a sequence of nuclear and budding cycles (Fig. 9). This ability of *Toxoplasma* and other parasites of this family to adapt their cell cycles to different hosts and tissues is one of the unsolved mysteries of Apicomplexa molecular biology. The key event that initiates the tachyzoite budding cycle is the duplication of the centrosome at the G1/S boundary [10,23]. Once duplicated, a complex process unfolds in and around these central structures, including assembly of the striated fiber required for budding initiation [15], formation of the spindle needed for mitosis, and organelle segregation [4]. How the centrosome performs the myriad of coordinating functions may be explained in part by our discovery of a unique binary internal organization. In this study, we show that the tachyzoite centrosome has two replicating core complexes. These cores have a distinct protein composition and a stereotypical geometry that defines their orientation with respect to the nucleus and forming daughter cells (Fig. 9, budding cycle). Interestingly, protein kinases, including TgArk1, TgMAPK-L1, and TgNek1–2 [54] appear to decorate specific structures in and around the centrosome indicating a complex and specialized regulatory machinery likely operates from the centrosome. The inner core complex (closest to the nucleus) of the tachyzoite centrosome contains factors TgCEP250 and TgCEP250-L1 and is aligned with the centrocone (Fig. 9), which in turn is oriented to the intranuclear kinetochore/centromeres throughout the cell cycle [25]. This arrangement suggests the inner core and centrocone may work in concert to control the adjacent nuclear environment. The unusual co-amplification of the centrocone and the inner centrosome core in the ts-TgSfi1 mutant shown here is consistent with a shared regulatory relationship. By contrast, the principle role for the centrosome outer core complex, which contains factors TgCentrin1/TgSfi1, TgSas-6, and the large TgArk1 protein kinase, is in regulating the initiation and assembly of daughter buds (Fig. 9, budding versus nuclear cycles). Genetic experiments strongly support this spatial segregation of function. The loss of the outer core in mutant 9–86E4, which we show in this study to be defective in ts-TgSfi1, leads to a primary block in budding (Fig. 6). Conversely, the Gubbels laboratory has recently produced a mutant of TgCEP250 leading to a loss of the inner core and a primary disruption of mitosis, while duplication of the outer core and budding initiation was not lost (Chen and Gubbels, personal communication). These consequences are reciprocal to the loss of budding and outer cores in the ts-TgSfi1 mutant; here the TgCEP250 inner core amplifies and the nucleus divides (Fig. 6). Altogether, these results indicate that the principle roles of the outer and inner centrosome cores in cytokinesis and karyokinesis, respectively, can be uncoupled. Notwithstanding the distinct protein composition and unusual independent function of the tachyzoite centrosome cores, there are other mechanisms that ensure each type of core is inherited by daughter parasites. Both cores duplicate at the G1/S phase transition, segregate by pairs into each daughter, and are co-regulated by factors residing in the PCM, which surrounds the internal cores. The loss of ts-TgMAPK-L1 in the temperature-sensitive mutant 11–31G12 leads to abnormal amplification of both centrosome cores and disrupts the normal linkage between the new daughter cytoskeleton and the centrocone structure (Fig. 7 and Fig. 8). Consistent with previous studies in which spindle and subpellicular microtubules were disrupted with oryzalin [28], breaking the physical connection between mitotic and budding machinery in the ts-TgMAPK-L1 mutant also causes a loss of normal restrictions over nuclear and daughter bud duplication. These results indicate TgMAPK-L1 has a key role in determining the scale of parasite counting in *Toxoplasma* replication (Fig. 9).

Proteomic analysis of human centrosomes reveals a more extensive asymmetrical protein distribution in centrosome structures than previously considered [59], and examples of this core diversity are elegantly demonstrated by recent high-resolution microscopy of human centriole structures [51]. Structural heterogeneity of the centrosome is thought to pave the way for specialized development [60] and also represents stages of procentriole maturation, although the diversity of novel protein complexes in centrosomes from different eukaryotes [60] suggests we are only beginning to understand the functions involved. The family history of the centrosome cores in the *T*. *gondii* tachyzoite is not fully understood; however, the spatiotemporal behavior of these structures is very distinct from animal cells. Coccidian parasites, which include *T*. *gondii*, possess recognizable centrioles in their centrosomes that are arranged not orthogonal, but in distinct parallel configuration [17,61]. They are also shaped differently (200 nm x 200 nm) and have a nine plus one all singlet organization [17,61]. There does not appear to be an equivalent procentriole maturation process as seen in animal cells; rather, we have shown here that the outer TgCentrin1/TgSfi-1 core duplicates first in early S phase, followed in minutes by the duplication of the TgCEP250/TgCEP250-L1 inner core. These core structures progressively resolve from each other over the next hour, reaching > 400 nm of separation in the post-metaphase of tachyzoite mitosis. Our results also demonstrate the TgCEP250/TgCEP250-L1 inner core never acquires outer core proteins TgCentrin1/TgSfi1, TgSas-6, or TgArk1 kinase. This would be expected as cells progress through successive cell cycles, if the inner core was a procentriole destined to become a mature mother centriole. These findings raise questions about the biogenesis of the tachyzoite centrosome core structures. The association of TgSas-6 cartwheel protein only with the outer TgCentrin1/TgSfi1 core provides a known template capable of seeding the duplication of this structure, as this factor templates the 9-fold symmetry of MT assembly of centrioles in other eukaryotes [30,35]. How the inner core replicates is more of a mystery because we did not detect TgSas-6 in this structure. It is possible we missed a transient association of TgSas-6 with the inner core, or de novo synthesis of the inner core centriole is responsible for duplication, which is known to occur in rare instances in eukaryotic cells [60]. Higher resolution studies of centrosome biogenesis in these parasites should help resolve these questions and test if both outer and inner centrosome cores in *Toxoplasma* tachyzoites have centrioles, although it remains a possibility that the inner core is part of the unique spindle pole complex, and therefore lacks a centriole.

Comprehensive phylogenetic analysis of several centriolar factors defined the inheritance of an ancestral module that regulates the 9-fold symmetry and the assembly of the centriole microtubules that precedes bikont and unikont divergence [37]. We have shown here that several of these core centrosome proteins are conserved in *T*. *gondii*, and they are also found encoded in the genomes of most other apicomplexans. It is therefore surprising that experimental evidence of the MT centriole barrels in the centrosome exists only for the coccidian branch, while the cartwheel protein Sas-6 that templates this structure is present in all apicomplexan branches (Table 1) [30,37]. Centrioles of *Toxoplasma* are small (200 x 200 nm) compared to animal cells (700 x 250 nm) and experimentally challenging to recognize in ultrastructure preparations [4]. Intriguingly, *P*. *falciparum* orthologs of several *T*. *gondii* outer core proteins have peak mRNA expression quite late in the intraerythrocytic cycle. Importantly, the mRNA encoding PfSas-6 cartwheel protein (PF3D7_0607600) is maximum at >35 h in growth-synchronized merozoites. This profile indicates that peak PfSas6 expression occurs in late schizogony, which is several hours after the initiation of nuclear replication in nuclear cycle [14]. PfSas-4 (PF3D7_1458500) and the ortholog of the large *T*. *gondii* aurora-related kinase (PF3D7_0309200) as well as one of two centrins (PfCEN2) that associate with the *P*. *falciparum* centrosome are also exclusively expressed in the late schizont (PfCEN2 and 3) [62]. The late expression of PfSas-6, PfCEN2, and the aurora-related kinase may indicate they function in the final cell cycle as part of the global control machinery that coordinates budding. Such a switch from local to global control is required to allow for synchronous budding and to complete *P*. *falciparum* merozoite replication [1]. Many of the molecular details of centrosome architecture and function in the Apicomplexa remain to be explored. However, the critical problem of how cytokinesis (budding) might be suspended during nuclear reduplication in the Apicomplexa may be solved by the fundamental independence of a two-compartment centrosome, in which one compartment controls budding while the other rules mitosis that we describe here for the *T*. *gondii* tachyzoite. This model provides a new framework to understand how multi-nuclear schizogony replication of *P*. *falciparum* and other important apicomplexan parasites is achieved. As noted previously [1], the unusually complex mitotic structures in these parasites appears to be a mix of strategic elements reminiscent of the mammalian extranuclear centriolar centrosome and the nuclear embedded yeast spindle pole body. This complex architecture may have evolved in these parasites to achieve regulatory diversity. Importantly, these distinct structural elements are connected and often remain physically tethered in a fixed linear array during the tachyzoite budding cycle. Spindle microtubules from the chromosomal centromeres hitch the genome to the inner core of the centrosome (note that centromeres remain sequestered in this region through interphase). Emanating from the centriolar region of the centrosome, the striated fiber connects to the new apical microtubule-organizing center of the daughter bud [15]. This fiber extends during the growth of the bud pellicle and then disappears as the daughter cytoskeleton reaches maturity. Given the elegant demonstration of active cyclin-CDK protein complexes tethered to the mitotic spindle in Hela cells [63], it is not a stretch to suggest that cell cycle checkpoints in the Apicomplexa likely exploit the remarkable physical connections from chromosome to daughter bud in order to coordinate cytokinesis and karyokinesis in the budding cycle. There are few examples of eukaryotic cells with specialized centrosome cores, and we would propose that this arrangement could achieve the regulatory flexibility required for these parasites to adapt to multiple host life cycles (Fig. 9). The implication of this model is that differential regulation of centrosome core composition and/or activity could provide the switch between the local control of chromosome replication in the nuclear cycle to the globally controlled "copy once" regimen of the budding cycle (Fig. 9). It is conceivable that post-translational regulation of the outer core could regulate activation or suspension of the budding cycle (Fig. 9, nuclear cycle), and the presence of TgArk1 exclusively in this core structure highlights a possible candidate regulator. Further studies to define how multiple organizational hubs (i.e., centrosome and centrocone) segregate responsibilities within the apicomplexan cell cycle will be important to understand this critical and truly fascinating aspect of the parasites’ life cycles.

## Materials and Methods

### Parasite Cell Culture

Parasites were grown in human foreskin fibroblasts (HFF) as described [64]. All transgenic and mutant parasite lines are derivatives of the RHΔ*hxgprt* parasite strain [65]. Temperature-sensitive clones 9–86E4 and 11–31G12 were obtained by chemical mutagenesis of the RHΔ*hxgprt* strain [21]. Growth measurements were performed using parasites pre-synchronized by limited invasion, as previously described [12,26]. Parasite vacuoles in the infected cultures were evaluated over various time periods with average vacuole sizes determined at each time point from 50–100 randomly selected vacuoles.

### Generation of Transgenic Tachyzoite Strains

**Endogenous tagging by genetic knock-in technique.** Selected *T*. *gondii* proteins were tagged with a triple copy of the HA or myc tag by genetic knock-in (See S2 Table for full list of genes, primers and transgenic strains created in the current study). PCR DNA fragments encompassing the 3′-end of the gene of interest (GOI) were used to construct the plasmids pLIC-GOI-HA_3X_/*dhfr*-DHFR-TS, pLIC-GOI-HA_3X_/*dhfr*-HXGPRT or pLIC-GOI-myc_3X_/*dhfr*-DHFR-TS and the constructs were electroporated into RHΔ*ku80* strain deficient in non-homologous recombination [66]. The double-tagged transgenic lines were established by sequential selection under alternative selection markers with cloning. Expression of the epitope tagged fusion proteins was verified by IFA.

A new strain expressing the ts-TgMAPK-L1 mutation was generated in the RHΔ*ku80* strain. To introduce the L534Q mutation into a new genomic background, we PCR amplified a 3,354 bp DNA fragment from mutant 11–31G12 that includes the 3′ end of the TGGT1_312570 (ts-TgMAPK-L1) using primers LIC-TgMAPK-L1_FOR and LIC-TgMAPK-L1_REV (S2 Table). In parallel, we also amplified a genomic fragment from the wild-type TGGT1_312570 locus in the RHΔ*ku80* to generate 3xHA tagged native TgMAPK-L1^HA^. The PCR products were cloned into pLIC-HA_3X_/*dhfr*-HXGPRT vector, and the resulting construct was introduced in RHΔ*ku80* strain [66]. Strains were tested for growth at 40°C and analyzed by IFA and western blot analysis.

**Endogenous tagging using CRISPR/Cas9 technology.** To introduce 3xHA-epitope to the C-terminus of ts-TgSfi1, we constructed gsTgSfi1 CRISPR/Cas9 plasmid by modifying sgUPRT-CRISPR/Cas9 plasmid generously provided by Dr. David Sibley (Washington University, MO, United States), as previously described [47]. Replacement was driven by Q5 DNA polymerase mutagenesis (New England Biolabs, Ipswich, MA, US) using primers specific for TgSfi1 gsRNA (S2 Table). To obtain the insertion cassette that includes C-terminus of TgSfi1 gene fused to 3xHA-epitope and HXGPRT selection marker we introduced synonymous mutation into the PAM site of the corresponding gsRNA in the pLIC-tgSfi1-HA_3X_/*dhfr*-HXGPRT plasmid using Q5 DNA polymerase mutagenesis (see primers design in the S2 Table). The amplified insertion cassette and sgTgSfi1 CRISPR/Cas9 plasmid were mixed in 1:1 molar ratio and electoporated into the mutant 9–86E4 parasites. Selection for growth in the mycophenolic acid/xanthine media was performed at 34°C.

**Ectopic expression of epitope-tagged TgCEP250-L1.** A large insert fosmid clone containing a fragment of chromosome IX (3757055–3790435) that includes the TgCEP250-L1 gene (RHfos10J10) [67] was modified by recombination with a cassette containing a 3xHA epitope tag, a chloramphenicol selection cassette (*T*. *gondii* selection), and a gentamycin selection cassette (for selection in bacteria) downstream of the TgCEP250-L1 gene. Primers contained appropriate overhangs to provide homology for recombination into the TgCEP250-L1 gene 3′end in the fosmid were used (S2 Table). Recombined fosmid was introduced in the mutants 9–86E4 and 11–31G12 and selected in the medium with 20 μM chloramphenicol. Established clones were analyzed by IFA and tested for growth at the permissive (34°C) and non-permissive (40°C) temperatures.

**Ectopic expression of epitope-tagged TgCentrin2.** The coding sequence of TgCentrin2 (TGME49_250340) was amplified from RHΔ*hxgprt* cDNA library (see primers design in the S2 Table) and cloned into the pDEST_tub-YFP_CAT plasmid by recombination (Gateway, Life Technologies), which resulted in the C-terminal fusion of TgCentrin2 with YFP-protein. Recombinant plasmid was introduced into RHΔ*ku80* parasites expressing endogenously tagged TgPAP^HA^ protein.

### Identification of Temperature-Sensitive Mutations

Temperature-sensitive mutants 9–86E4 (ts-TgSfi1) and 11–31G12 (ts-TgMAPK-L1) were complemented using the ToxoSuperCos cosmid genomic library as previously described [21,23,26]. Mutant parasites were transfected with cosmid library DNA (50 μg DNA/5 x 10^7^ parasites/transfection) in 20 independent electroporations. After two consecutive selections at 40°C, parasites were selected by the combination of high temperature and 1 μM pyrimethamine. Double-resistant (temperature and drug) populations were passed four times before genomic DNA was isolated for marker-rescue [21]. To identify the complementing locus in *T*. *gondii* chromosomes, rescued genomic inserts were sequenced using a T3 primer and the sequences mapped to the *T*. *gondii* genome (Toxodb). To resolve the contribution of individual genes in the recovered locus, we transformed the mutants with individual cosmids from a cosmid collection mapped to the *T*. *gondii* genome (toxomap.wustl.edu/cosmid.html). For direct complementation of the mutant 11–31G12 with DNA fragments, the TGGT1_312570 gene locus (including 550 bp 5′UTR and 367 bp of 3′UTR) was amplified from genomic DNA isolated from the parental strain RHΔ*hxgprt* or the mutant (S2 Table). Specific cosmids or PCR fragments were transfected into 1 x 10^7^ parasites using 6–10 μg of purified DNA. To quantify genetic rescue, established drug-resistant populations were tested for growth at the high temperature by standard plaque assay performed in triplicate [21,23,26]

To validate the cosmid genetic complementation we used next generation sequencing to verify the mutation in mutant 9–86E4 (ts-TgSfi1). Whole genome DNA libraries were prepared, sequenced, and analyzed for single nucleotide variation (SNV) according to published methods [68]. In brief, genomic DNA from the mutant and parent strains was fragmented and then, following end-repair, ligated with Illumina paired-end adaptors (Illumina, CA, US). Purified library fragments were enriched via PCR amplification using Illumina paired-end PCR primers (Illumina, CA, US), the fragments normalized to 2 nM and denatured using 0.1 N NaOH. Denatured libraries were cluster amplified on V2 flowcells using V2 chemistry according to manufacturer’s protocol (Illumina, CA, US). Flowcells were sequenced on Genome Analyzer II’s, using V3 Sequencing-by-Synthesis kits and analyzed with the Illumina’s v1.3.4 pipeline following manufacturer’s protocol (Illumina, CA, US). The resulting FASTQ sequence traces were aligned to *T*. *gondii* GT1 genome reference v7.3 and the Human genome reference build 37. MOSAIK was used to perform the alignments using the standard parameters described in the documentation V1.0 (available at https://github.com/wanpinglee/MOSAIK/wiki/QuickStart). SNVs were called using the SNV caller FreeBayes, using standard parameters as described in the documentation, software version 0.7.2. SNVs were filtered to remove SNVs whose calls had less than 5x coverage in the mutant and 3x in the parent, a *p*-value less than 0.8, and did not have a single allele that comprised 70% or more of the sequence reads.

### Western Blot Analysis

Purified parasites were washed in PBS and collected by centrifugation. Total lysates were obtained by resuspending the parasite pellets with Leammli loading dye, heated at 95°C for 10 min, and briefly sonicated. After separation on the SDS-PAGE gels, proteins were transferred onto nitrocellulose membrane and probed with monoclonal antibodies against HA- (rat 3F10, Roche Applied Sciences), myc-epitope (mouse, Cell Signaling Technology), and α-Tubulin (mouse 12G10, kindly provided by Dr. Jacek Gaertig, University of Georgia, GA, US). After incubation with secondary HRP-conjugated anti-mouse or anti-rat antibodies, proteins were visualized by enhanced chemiluminescence detection (PerkinElmer).

### Immunofluorescence Analysis

Confluent HFF cultures on glass coverslips were infected with parasites for the indicated times. Infected monolayers were fixed, permeabilized, and incubated with antibody as previously described [26]. The following primary antibodies were used: mouse monoclonal αMyc (Santa Cruz Biotechnology, Santa Cruz, CA, US), αTgCenH3 [25], rat monoclonal αHA (Roche Applied Sciences), rabbit polyclonal αMyc (Cell Signaling Technology), αHuman Centrin 2 [23], αMORN1 (centrocone and basal complex stains, kindly provided by Dr. Marc-Jan Gubbels, Boston College, MA, US), and αIMC1 (parasite shape and internal daughter bud stains, kindly provided by Dr. Gary Ward, University of Vermont, VT, US). All Alexa-conjugated secondary antibodies (Molecular Probes, Life Technologies) were used at dilution 1:500. Coverslips were mounted with Aquamount (Thermo Scientific), dried overnight at 4°C, and viewed on Zeiss Axiovert Microscope equipped with 100x objective. Images were processed in Adobe Photoshop CS v4.0 using linear adjustment when needed. Super-Resolution images were acquired using the Zeiss ELYRA S1 (SR-SIM) microscope using a 63x lens. Images were collected and processed using Zeiss Zen software.

## Supporting Information

S1 Data Raw data plotted in Figs. 5, 6, 7, and 8. (XLSX)

S1 Fig *T*. *gondii* centrosomal proteins are structurally similar to the human orthologs. (A and D) Coiled-coil domains of *T*. *gondii* (blue) and human (red) orthologs predicted using Marcoil algorithm (http://toolkit.tuebingen.mpg.de/marcoil) are shown. Extended coiled-coils longer than 200 amino acids are shown in the diagram above each corresponding plot, along with the additional detected domains as well as conservation at the primary sequence level. (B) Western blot analysis of *Toxoplasma* proteins epitope-tagged in the current study. Observed molecular weight of the proteins correlated with the predicted protein masses: TgSas-6^myc^, 136 kDa; TgCEP250-L1^myc^, 301 kDa; Tgγ-Tubulin^myc^, 56 kDa; TgSfi1^myc^, 434 kDa; TgNMP1^myc^, 159 kDa; TgPAP1^HA^, 202 kDa; TgArk1^HA^, 302 kDa; TgCentrin1^HA^, 23 kDa. Equal loading of parasite lysate was confirmed by counter stain with the anti-αTubulin antibody. (C) High levels of disorder were detected in the TgSfi1 structure (>0.5, dashed line) using PONDR algorithm (http://www.pondr.com). Diagram on the top shows predicted centrin-binding motifs and the region of conservation with the human Sfi1 ortholog.
(TIF)

S2 Fig TgSfi1 predicted centrin-binding repeats. The predicted protein sequence for TgSfi1 is shown with divergent centrin-binding sites highlighted in the sequence. The centrin-binding consensus motif determined from yeast and human Sfi1 orthologs is shown on the top [38]. Note that centrin-binding is predicted to span the N-terminal, 2/3 of the total protein length.
(TIF)

S3 Fig Two CEP250-related proteins have distinct localization (not centrosomal) in the *T*. *gondii* tachyzoite stage. (A and B) Structural features of two novel coiled-coil domain proteins with similarity to TgCEP250 as predicted by the Marcoil algorithm (http://toolkit.tuebingen.mpg.de/marcoil). The coiled-coil domains are indicated above each graph. The localization patterns for each protein are shown: (A) TGME49_242790 (TgPAP1) is localized in the peripheral annuli, which is a compartment within the newly forming daughter parasites previously identified in the study of Hu et al. [43], and (B) TGME49_265840 (TgNMP1) was localized to the perinuclear meshwork. In each transgenic parasite studied here, the protein of interest was tagged by genetic knock-in into the endogenous locus with a triple copy of the HA epitope resulting in a C-terminal protein fusion [66]. To confirm peripheral annuli localization, the TgPAP1^HA^ protein was co-localized with the compartment marker TgCentrin2-YFP (bottom images on panel A). The white arrow indicates the apical cluster of peripheral annuli where TgPAP1 co-localize with TgCentrin2. TgCentrin2, additionally, occupies the centrosome (white arrow head only) and the conoid (green arrow head).
(TIF)

S4 Fig TgMAPK-L1 is an essential mitotic factor in *T*. *gondii*. (A) Dynamic cell cycle expression of the endogenously tagged TgMAPK-L1^HA^ was revealed by co-staining with inner membrane protein (IMC1, red) and nuclear dye DAPI (blue). Strong expression of TgMAPK-L1 in the perinuclear region was detected in S-phase (S: second panel). After transient translocation to the newly forming daughter bud (M/C: third panel) TgMAPK-L1 gradually declined in cytokinesis (C: bottom panel). Cell cycle phases of individual parasites and vacuoles were determined based on well-established nuclear and cell morphological criteria [4]. (B) Genetic complementation of mutant 11–31G12 with cosmid genomic libraries identified a defective locus on the chromosome XI spanning three possible genes: TGGT1_312560 (gene 1), TGGT1_312570 (gene 2), and TGGT1_312580 (gene 3). Secondary complementation with cosmids spanning the locus (**tiled cosmids**) identified gene 3 encoding a putative CGMC kinase (TgMAPK-L1) as responsible for the ts-defect. The finding was further confirmed by complementation of mutant 11–31G12 with amplified genomic fragments (**transformed cDNA**) spanning the wild type or ts-allele. (C) Schematic of TgMAPK-L1 features shows the location of ts-mutation and the corresponding change in amino acid residue. (D) Strategy for introduction of the ts-TgMAPK-L1 mutation (L534Q) by genetic knock-in while simultaneously tagging the gene with 3xHA in the RHΔ*ku80* strain. (E) ts-TgMAPK-L1^HA^ (green) localized to the centrosomal region of dividing parasites (34°C) and in high temperature arrested parasites ts-TgMAPK-L1 was degraded (40°C). (F) Instability of the ts-TgMAPK-L1 at 40°C appears to be a major contributing factor to the growth arrest of mutant parasites at high temperature. Total lysates of RHΔ*ku80* expressing ts-TgMAPK-L1^HA^ grown for 24 h at 34°C or 40°C were analyzed by western blotting and probed with anti-HA antibody. Equal loading of parasite lysate was confirmed by counter stains with the anti-αTubulin antibody.
(TIF)

S1 Table *T*. *gondii* CEP250-related proteins with coiled-coil domains. (DOCX)

S2 Table Transgenic *T*. *gondii* strains and primers used in the study. (XLSX)

## Abbreviations

CEP: CEntrosomal Proteins
M/C: completion of mitosis and budding
MTs: microtubules
MTOC: microtubule-organizing center
PCM: pericentriolar matrix
PISA: Present in Sas-6

## References

[1] Arnot DE, Ronander E, Bengtsson DC (2011) The progression of the intra-erythrocytic cell cycle of Plasmodium falciparum and the role of the centriolar plaques in asynchronous mitotic division during schizogony. Int J Parasitol 41: 71–80. 10.1016/j.ijpara.2010.07.012 20816844

[2] Sogin ML, Silberman JD (1998) Evolution of the protists and protistan parasites from the perspective of molecular systematics. Int J Parasitol 28: 11–20. 9504331 10.1016/s0020-7519(97)00181-1

[3] Anderson-White B, Beck JR, Chen CT, Meissner M, Bradley PJ, et al (2012) Cytoskeleton assembly in Toxoplasma gondii cell division. International review of cell and molecular biology 298: 1–31. 10.1016/B978-0-12-394309-5.00001-8 22878103 PMC4066374

[4] Francia ME, Striepen B (2014) Cell division in apicomplexan parasites. Nat Rev Microbiol 12: 125–136. 10.1038/nrmicro3184 24384598

[5] White MW, Radke JR, Radke JB (2014) Toxoplasma development—turn the switch on or off? Cell Microbiol 16: 466–472. 10.1111/cmi.12267 24438211

[6] Ferguson DJ, Dubremetz JF (2014) The Ultrastructure of Toxoplasma gondii In: Weiss LM, Kim K, editors. Toxoplasma gondii The model Apicomplexan: Perspectives and Methods. second ed: Elsevier pp. 1085.

[7] Behnke MS, Wootton JC, Lehmann MM, Radke JB, Lucas O, et al (2010) Coordinated progression through two subtranscriptomes underlies the tachyzoite cycle of Toxoplasma gondii. PLoS ONE 5: e12354 10.1371/journal.pone.0012354 20865045 PMC2928733

[8] Radke JR, Striepen B, Guerini MN, Jerome ME, Roos DS, et al (2001) Defining the cell cycle for the tachyzoite stage of Toxoplasma gondii. Mol Biochem Parasitol 115: 165–175. 11420103 10.1016/s0166-6851(01)00284-5

[9] Radke JR, White MW (1998) A cell cycle model for the tachyzoite of *Toxoplasma gondii* using the Herpes simplex virus thymidine kinase. Mol Biochem Parasitol 94: 237–247. 9747974 10.1016/s0166-6851(98)00074-7

[10] White MW, Conde de Felipe M, Lehmann M, Radke JR (2007) Cell cycle control and parasite division.; Aijoka JW, Soldati D, editors. Norwich, UK: Horizon Scientific Press.

[11] Conde de Felipe MM, Lehmann MM, Jerome ME, White MW (2008) Inhibition of Toxoplasma gondii growth by pyrrolidine dithiocarbamate is cell cycle specific and leads to population synchronization. Mol Biochem Parasitol 157: 22–31. 17976834 10.1016/j.molbiopara.2007.09.003 PMC2222652

[12] Gaji RY, Behnke MS, Lehmann MM, White MW, Carruthers VB (2011) Cell cycle-dependent, intercellular transmission of Toxoplasma gondii is accompanied by marked changes in parasite gene expression. Molecular microbiology 79: 192–204. 10.1111/j.1365-2958.2010.07441.x 21166903 PMC3075969

[13] Nishi M, Hu K, Murray JM, Roos DS (2008) Organellar dynamics during the cell cycle of Toxoplasma gondii. J Cell Sci 121: 1559–1568. 10.1242/jcs.021089 18411248 PMC6810632

[14] Bozdech Z, Llinas M, Pulliam BL, Wong ED, Zhu J, et al (2003) The transcriptome of the intraerythrocytic developmental cycle of Plasmodium falciparum. PLoS Biol 1: E5 12929205 10.1371/journal.pbio.0000005 PMC176545

[15] Francia ME, Jordan CN, Patel JD, Sheiner L, Demerly JL, et al (2012) Cell division in Apicomplexan parasites is organized by a homolog of the striated rootlet fiber of algal flagella. PLoS Biol 10: e1001444 10.1371/journal.pbio.1001444 23239939 PMC3519896

[16] Dubremetz JF (1973) [Ultrastructural study of schizogonic mitosis in the coccidian, Eimeria necatrix (Johnson 1930)]. J Ultrastruct Res 42: 354–376. 4702924

[17] Dubremetz JF (1975) [Genesis of merozoites in the coccidia, Eimeria necatrix. Ultrastructural study]. J Protozool 22: 71–84. 1117438 10.1111/j.1550-7408.1975.tb00946.x

[18] Dubremetz JF, Elsner YY (1979) Ultrastructural study of schizogony of Eimeria bovis in cell cultures. J Protozool 26: 367–376. 536929 10.1111/j.1550-7408.1979.tb04639.x

[19] Agop-Nersesian C, Egarter S, Langsley G, Foth BJ, Ferguson DJ, et al (2010) Biogenesis of the inner membrane complex is dependent on vesicular transport by the alveolate specific GTPase Rab11B. PLoS Pathog 6: e1001029 10.1371/journal.ppat.1001029 20686666 PMC2912401

[20] Hu K, Mann T, Striepen B, Beckers CJ, Roos DS, et al (2002) Daughter Cell Assembly in the Protozoan Parasite Toxoplasma gondii. Mol Biol Cell 13: 593–606. 11854415 10.1091/mbc.01-06-0309 PMC65652

[21] Gubbels MJ, Lehmann M, Muthalagi M, Jerome ME, Brooks CF, et al (2008) Forward genetic analysis of the apicomplexan cell division cycle in Toxoplasma gondii. PLoS Pathog 4: e36 10.1371/journal.ppat.0040036 18282098 PMC2242837

[22] Gubbels MJ, White M, Szatanek T (2008) The cell cycle and Toxoplasma gondii cell division: tightly knit or loosely stitched? Int J Parasitol 38: 1343–1358. 10.1016/j.ijpara.2008.06.004 18703066

[23] Suvorova ES, Radke JB, Ting LM, Vinayak S, Alvarez CA, et al (2013) A nucleolar AAA-NTPase is required for parasite division. Mol Microbiol 90: 338–355. 10.1111/mmi.12367 23964771 PMC3902653

[24] Ferguson DJ, Sahoo N, Pinches RA, Bumstead JM, Tomley FM, et al (2008) MORN1 has a conserved role in asexual and sexual development across the apicomplexa. Eukaryotic cell 7: 698–711. 10.1128/EC.00021-08 18310354 PMC2292627

[25] Brooks CF, Francia ME, Gissot M, Croken MM, Kim K, et al (2011) Toxoplasma gondii sequesters centromeres to a specific nuclear region throughout the cell cycle. Proceedings of the National Academy of Sciences of the United States of America 108: 3767–3772. 10.1073/pnas.1006741108 21321216 PMC3048097

[26] Suvorova ES, Lehmann MM, Kratzer S, White MW (2012) Nuclear actin-related protein is required for chromosome segregation in Toxoplasma gondii. Molecular and biochemical parasitology 181: 7–16. 10.1016/j.molbiopara.2011.09.006 21963440 PMC3767130

[27] White MW, Jerome ME, Vaishnava S, Guerini M, Behnke M, et al (2005) Genetic rescue of a Toxoplasma gondii conditional cell cycle mutant. Molecular microbiology 55: 1060–1071. 15686554 10.1111/j.1365-2958.2004.04471.x

[28] Morrissette NS, Sibley LD (2002) Disruption of microtubules uncouples budding and nuclear division in Toxoplasma gondii. J Cell Sci 115: 1017–1025. 11870220 10.1242/jcs.115.5.1017

[29] Gubbels MJ, Vaishnava S, Boot N, Dubremetz JF, Striepen B (2006) A MORN-repeat protein is a dynamic component of the Toxoplasma gondii cell division apparatus. Journal of cell science 119: 2236–2245. 16684814 10.1242/jcs.02949

[30] van Breugel M, Hirono M, Andreeva A, Yanagisawa HA, Yamaguchi S, et al (2011) Structures of SAS-6 suggest its organization in centrioles. Science 331: 1196–1199. 10.1126/science.1199325 21273447

[31] de Leon JC, Scheumann N, Beatty W, Beck JR, Tran JQ, et al (2013) A SAS-6-like protein suggests that the Toxoplasma conoid complex evolved from flagellar components. Eukaryot Cell 12: 1009–1019. 10.1128/EC.00096-13 23687115 PMC3697468

[32] Kirkham M, Muller-Reichert T, Oegema K, Grill S, Hyman AA (2003) SAS-4 is a C. elegans centriolar protein that controls centrosome size. Cell 112: 575–587. 12600319 10.1016/s0092-8674(03)00117-x

[33] Salisbury JL (2003) Centrosome size is controlled by centriolar SAS-4. Trends Cell Biol 13: 340–343. 12837604 10.1016/s0962-8924(03)00126-0

[34] Salisbury JL (2004) Centrosomes: Sfi1p and centrin unravel a structural riddle. Curr Biol 14: R27–29. 14711432 10.1016/j.cub.2003.12.019

[35] Kitagawa D, Vakonakis I, Olieric N, Hilbert M, Keller D, et al (2011) Structural basis of the 9-fold symmetry of centrioles. Cell 144: 364–375. 10.1016/j.cell.2011.01.008 21277013 PMC3089914

[36] Nakazawa Y, Hiraki M, Kamiya R, Hirono M (2007) SAS-6 is a cartwheel protein that establishes the 9-fold symmetry of the centriole. Curr Biol 17: 2169–2174. 18082404 10.1016/j.cub.2007.11.046

[37] Carvalho-Santos Z, Machado P, Branco P, Tavares-Cadete F, Rodrigues-Martins A, et al (2010) Stepwise evolution of the centriole-assembly pathway. J Cell Sci 123: 1414–1426. 10.1242/jcs.064931 20392737

[38] Kilmartin JV (2003) Sfi1p has conserved centrin-binding sites and an essential function in budding yeast spindle pole body duplication. J Cell Biol 162: 1211–1221. 14504268 10.1083/jcb.200307064 PMC2173958

[39] Obradovic Z, Peng K, Vucetic S, Radivojac P, Dunker AK (2005) Exploiting heterogeneous sequence properties improves prediction of protein disorder. Proteins 61 Suppl 7: 176–182. 16187360 10.1002/prot.20735

[40] Kumar A, Rajendran V, Sethumadhavan R, Purohit R (2013) CEP proteins: the knights of centrosome dynasty. Protoplasma 250: 965–983. 10.1007/s00709-013-0488-9 23456457

[41] Azimzadeh J, Bornens M (2007) Structure and duplication of the centrosome. J Cell Sci 120: 2139–2142. 17591686 10.1242/jcs.005231

[42] Gould SB, Kraft LG, van Dooren GG, Goodman CD, Ford KL, et al (2011) Ciliate pellicular proteome identifies novel protein families with characteristic repeat motifs that are common to alveolates. Mol Biol Evol 28: 1319–1331. 10.1093/molbev/msq321 21127172

[43] Hu K, Johnson J, Florens L, Fraunholz M, Suravajjala S, et al (2006) Cytoskeletal components of an invasion machine—the apical complex of Toxoplasma gondii. PLoS Pathog 2: e13 16518471 10.1371/journal.ppat.0020013 PMC1383488

[44] Azimzadeh J, Marshall WF (2010) Building the centriole. Curr Biol 20: R816–825. 10.1016/j.cub.2010.08.010 20869612 PMC2956124

[45] Brown KM, Suvorova E, Farrell A, McLain A, Dittmar A, et al (2014) Forward Genetic Screening Identifies a Small Molecule That Blocks Toxoplasma gondii Growth by Inhibiting Both Host- and Parasite-Encoded Kinases. PLoS Pathog 10: e1004180 10.1371/journal.ppat.1004180 24945800 PMC4055737

[46] Szatanek T, Anderson-White BR, Faugno-Fusci DM, White M, Saeij JP, et al (2012) Cactin is essential for G1 progression in Toxoplasma gondii. Molecular microbiology 84: 566–577. 10.1111/j.1365-2958.2012.08044.x 22486860 PMC3331927

[47] Shen B, Brown KM, Lee TD, Sibley LD (2014) Efficient gene disruption in diverse strains of Toxoplasma gondii using CRISPR/CAS9. MBio 5: e01114–01114. 10.1128/mBio.01114-14 24825012 PMC4030483

[48] Martinez-Sanz J, Kateb F, Assairi L, Blouquit Y, Bodenhausen G, et al (2010) Structure, dynamics and thermodynamics of the human centrin 2/hSfi1 complex. J Mol Biol 395: 191–204. 10.1016/j.jmb.2009.10.041 19857500

[49] Dorin-Semblat D, Quashie N, Halbert J, Sicard A, Doerig C, et al (2007) Functional characterization of both MAP kinases of the human malaria parasite Plasmodium falciparum by reverse genetics. Mol Microbiol 65: 1170–1180. 17651389 10.1111/j.1365-2958.2007.05859.x

[50] Carvalho T, Doerig C, Reininger L (2013) Nima- and Aurora-related kinases of malaria parasites. Biochim Biophys Acta 1834: 1336–1345. 10.1016/j.bbapap.2013.02.022 23462523

[51] Sonnen KF, Schermelleh L, Leonhardt H, Nigg EA (2012) 3D-structured illumination microscopy provides novel insight into architecture of human centrosomes. Biol Open 1: 965–976. 10.1242/bio.20122337 23213374 PMC3507176

[52] Roskoski R Jr. (2012) ERK1/2 MAP kinases: structure, function, and regulation. Pharmacol Res 66: 105–143. 10.1016/j.phrs.2012.04.005 22569528

[53] Ma HT, Poon RY (2011) How protein kinases co-ordinate mitosis in animal cells. Biochem J 435: 17–31. 10.1042/BJ20100284 21406064

[54] Chen CT, Gubbels MJ (2013) The Toxoplasma gondii centrosome is the platform for internal daughter budding as revealed by a Nek1 kinase mutant. J Cell Sci 126: 3344–3355. 10.1242/jcs.123364 23729737 PMC3730244

[55] Hochegger H, Hegarat N, Pereira-Leal JB (2013) Aurora at the pole and equator: overlapping functions of Aurora kinases in the mitotic spindle. Open Biol 3: 120185 10.1098/rsob.120185 23516109 PMC3718339

[56] Carvalho TG, Doerig C, Reininger L (2013) Nima- and Aurora-related kinases of malaria parasites. Biochim Biophys Acta 1834: 1336–1345. 10.1016/j.bbapap.2013.02.022 23462523

[57] Reininger L, Wilkes JM, Bourgade H, Miranda-Saavedra D, Doerig C (2011) An essential Aurora-related kinase transiently associates with spindle pole bodies during Plasmodium falciparum erythrocytic schizogony. Mol Microbiol 79: 205–221. 10.1111/j.1365-2958.2010.07442.x 21166904 PMC3025120

[58] Lorestani A, Ivey FD, Thirugnanam S, Busby MA, Marth GT, et al (2012) Targeted proteomic dissection of Toxoplasma cytoskeleton sub-compartments using MORN1. Cytoskeleton 69: 1069–1085. 10.1002/cm.21077 23027733 PMC3566231

[59] Jakobsen L, Vanselow K, Skogs M, Toyoda Y, Lundberg E, et al (2011) Novel asymmetrically localizing components of human centrosomes identified by complementary proteomics methods. EMBO J 30: 1520–1535. 10.1038/emboj.2011.63 21399614 PMC3102290

[60] Debec A, Sullivan W, Bettencourt-Dias M (2010) Centrioles: active players or passengers during mitosis? Cell Mol Life Sci 67: 2173–2194. 10.1007/s00018-010-0323-9 20300952 PMC2883084

[61] Sibert GJ, Speer CA (1981) Fine structure of nuclear division and microgametogony of Eimeria nieschulzi Dieben, 1924. Z Parasitenkd 66: 179–189. 7324546 10.1007/BF00925725

[62] Mahajan B, Selvapandiyan A, Gerald NJ, Majam V, Zheng H, et al (2008) Centrins, cell cycle regulation proteins in human malaria parasite Plasmodium falciparum. J Biol Chem 283: 31871–31883. 10.1074/jbc.M800028200 18693242

[63] Hagting A, Karlsson C, Clute P, Jackman M, Pines J (1998) MPF localization is controlled by nuclear export. EMBO J 17: 4127–4138. 9670027 10.1093/emboj/17.14.4127 PMC1170745

[64] Roos DS, Donald RG, Morrissette NS, Moulton AL (1994) Molecular tools for genetic dissection of the protozoan parasite Toxoplasma gondii. Methods in cell biology 45: 27–63. 7707991 10.1016/s0091-679x(08)61845-2

[65] Donald RG, Roos DS (1998) Gene knock-outs and allelic replacements in Toxoplasma gondii: HXGPRT as a selectable marker for hit-and-run mutagenesis. Mol Biochem Parasitol 91: 295–305. 9566522 10.1016/s0166-6851(97)00210-7

[66] Huynh MH, Carruthers VB (2009) Tagging of endogenous genes in a Toxoplasma gondii strain lacking Ku80. Eukaryot Cell 8: 530–539. 10.1128/EC.00358-08 19218426 PMC2669203

[67] Vinayak S, Brooks CF, Naumov A, Suvorova ES, White MW, et al (2014) Genetic manipulation of the Toxoplasma gondii genome by fosmid recombineering. MBio 5: e02021 10.1128/mBio.02021-14 25467441 PMC4324243

[68] Farrell A, Thirugnanam S, Lorestani A, Dvorin JD, Eidell KP, et al (2012) A DOC2 protein identified by mutational profiling is essential for apicomplexan parasite exocytosis. Science 335: 218–221. 10.1126/science.1210829 22246776 PMC3354045

