# Supplementary figures and images for: A Novel Bipartite Centrosome Coordinates the Apicomplexan Cell Cycle
Source: PLoS Biol. 2015 Mar 3;13(3):e1002093. doi: 10.1371/journal.pbio.1002093 (PMC4348508; doi:10.1371/journal.pbio.1002093)

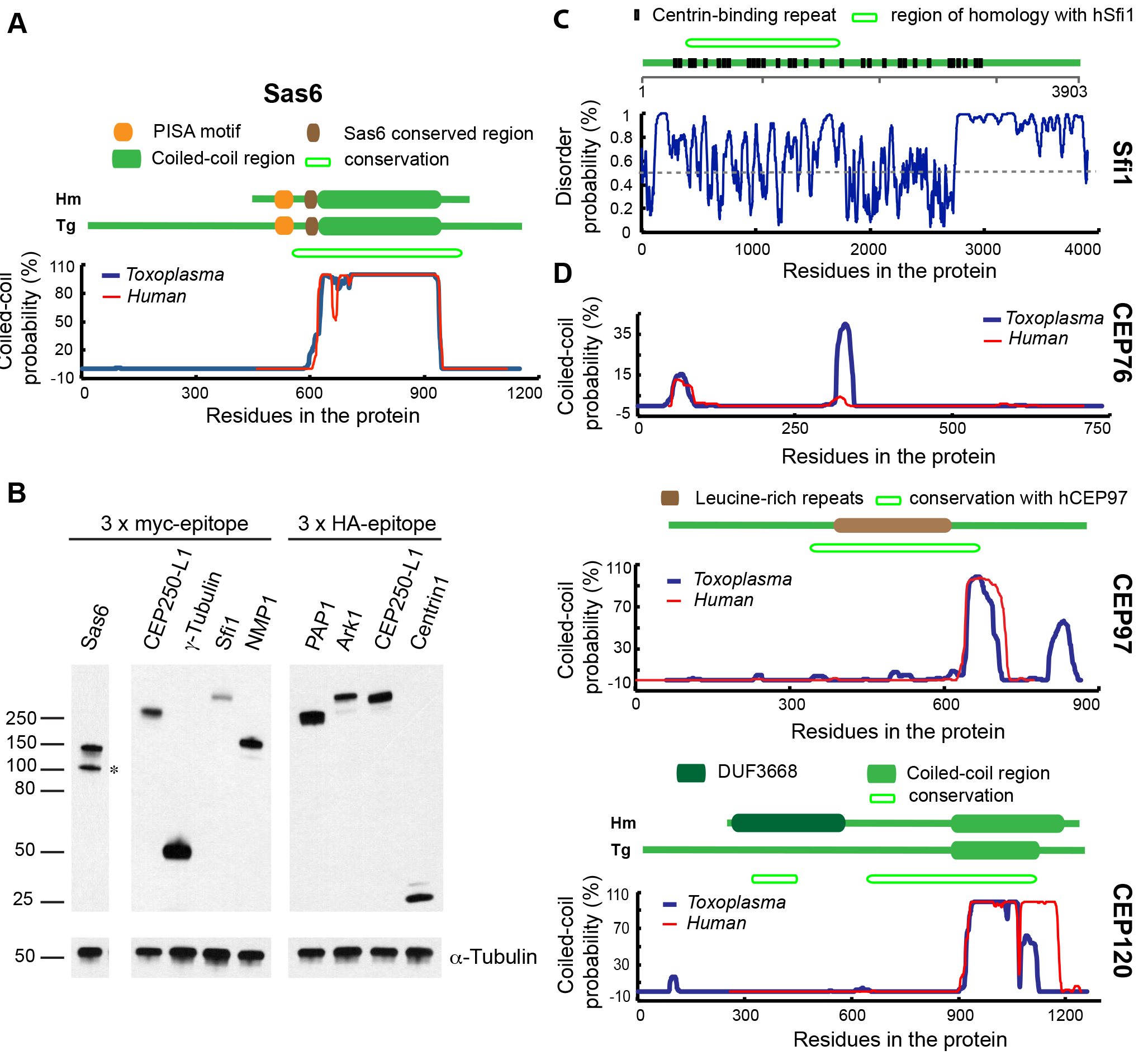

Supplement: S1 Fig — (A and D) Coiled-coil domains of T. gondii (blue) and human (red) orthologs predicted using Marcoil algorithm (http://toolkit.tuebingen.mpg.de/marcoil) are shown. Extended coiled-coils longer than 200 amino acids are shown in the diagram above each corresponding plot, along with the additional detected domains as well as conservation at the primary sequence level. (B) Western blot analysis of Toxoplasma proteins epitope-tagged in the current study. Observed molecular weight of the proteins correlated with the predicted protein masses: TgSas-6myc, 136 kDa; TgCEP250-L1myc, 301 kDa; Tgγ-Tubulinmyc, 56 kDa; TgSfi1myc, 434 kDa; TgNMP1myc, 159 kDa; TgPAP1HA, 202 kDa; TgArk1HA, 302 kDa; TgCentrin1HA, 23 kDa. Equal loading of parasite lysate was confirmed by counter stain with the anti-αTubulin antibody. (C) High levels of disorder were detected in the TgSfi1 structure (>0.5, dashed line) using PONDR algorithm (http://www.pondr.com). Diagram on the top shows predicted centrin-binding motifs and the region of conservation with the human Sfi1 ortholog. (TIF) [file pbio.1002093.s002.tif]

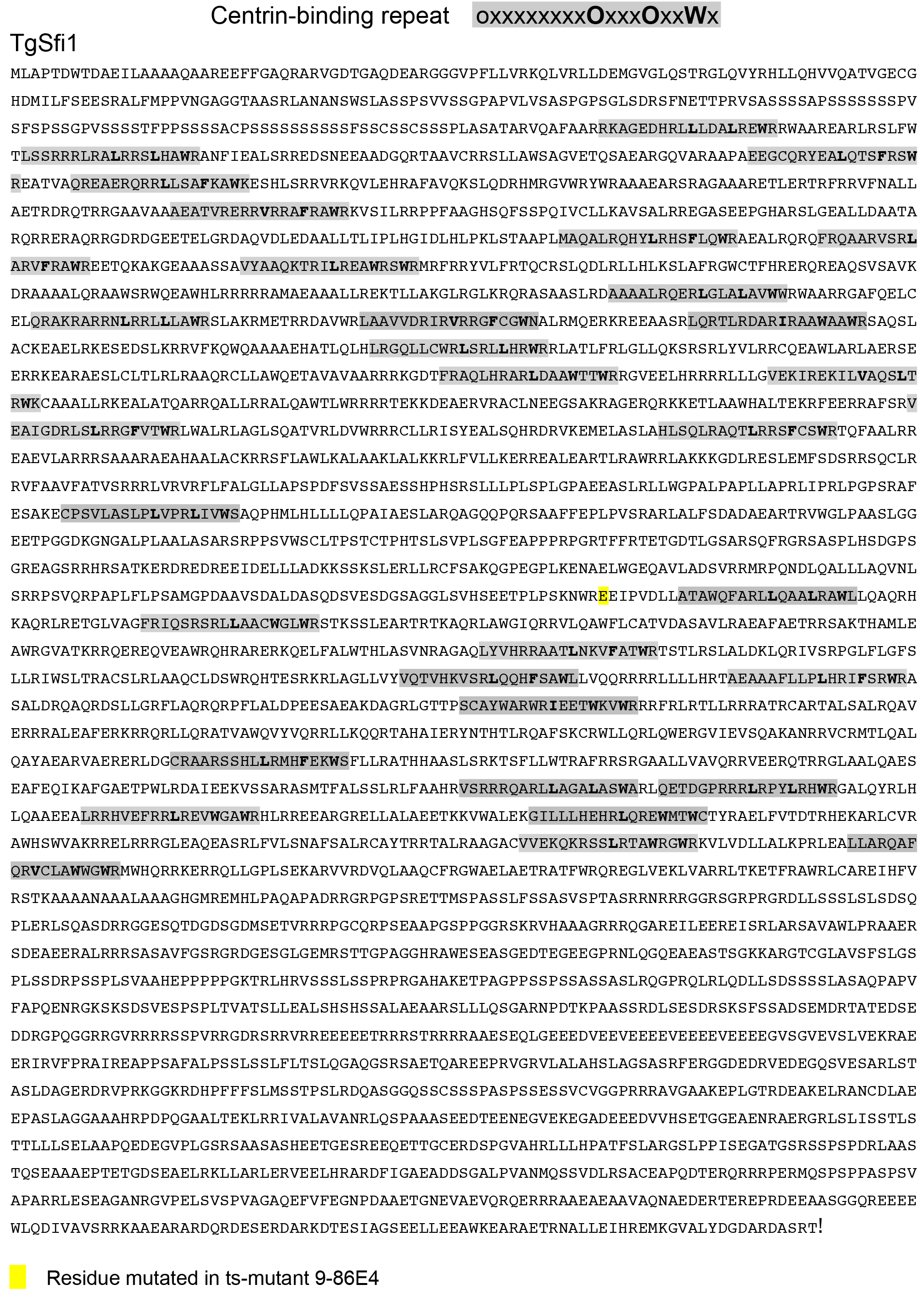

Supplement: S2 Fig — The predicted protein sequence for TgSfi1 is shown with divergent centrin-binding sites highlighted in the sequence. The centrin-binding consensus motif determined from yeast and human Sfi1 orthologs is shown on the top [38]. Note that centrin-binding is predicted to span the N-terminal, 2/3 of the total protein length. (TIF) [file pbio.1002093.s003.tif]

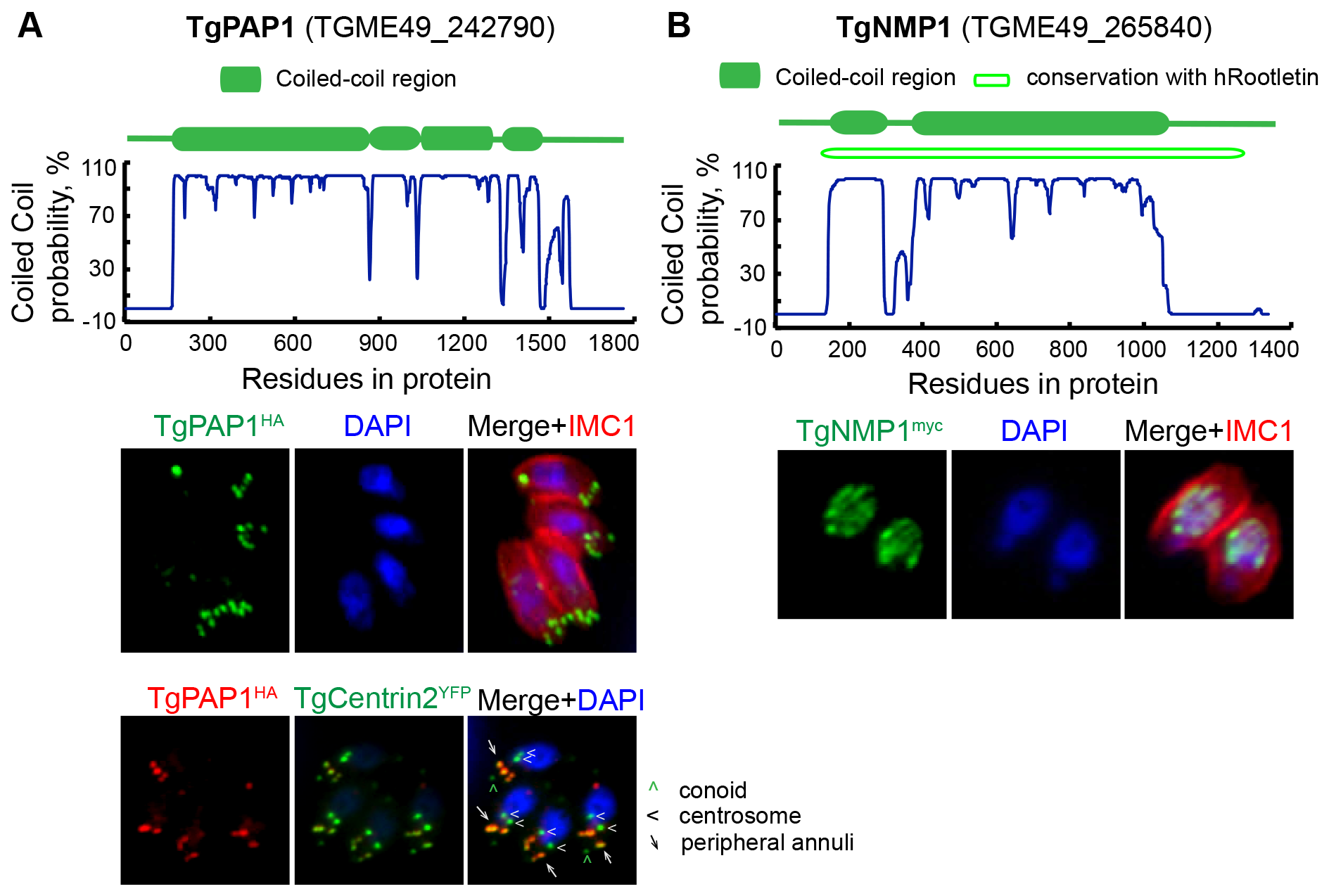

Supplement: S3 Fig — (A and B) Structural features of two novel coiled-coil domain proteins with similarity to TgCEP250 as predicted by the Marcoil algorithm (http://toolkit.tuebingen.mpg.de/marcoil). The coiled-coil domains are indicated above each graph. The localization patterns for each protein are shown: (A) TGME49_242790 (TgPAP1) is localized in the peripheral annuli, which is a compartment within the newly forming daughter parasites previously identified in the study of Hu et al. [43], and (B) TGME49_265840 (TgNMP1) was localized to the perinuclear meshwork. In each transgenic parasite studied here, the protein of interest was tagged by genetic knock-in into the endogenous locus with a triple copy of the HA epitope resulting in a C-terminal protein fusion [66]. To confirm peripheral annuli localization, the TgPAP1HA protein was co-localized with the compartment marker TgCentrin2-YFP (bottom images on panel A). The white arrow indicates the apical cluster of peripheral annuli where TgPAP1 co-localize with TgCentrin2. TgCentrin2, additionally, occupies the centrosome (white arrow head only) and the conoid (green arrow head). (TIF) [file pbio.1002093.s004.tif]

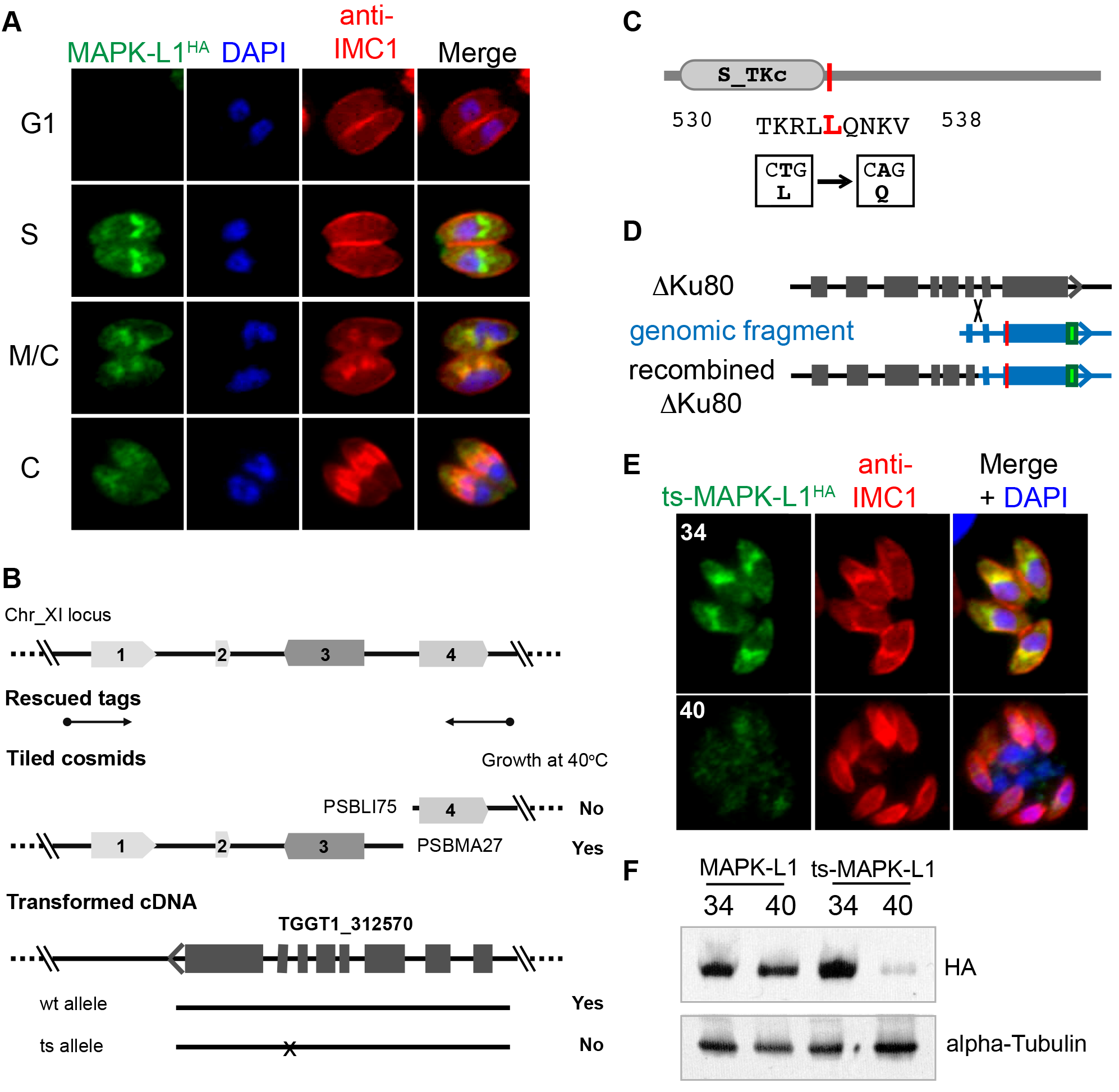

Supplement: S4 Fig — (A) Dynamic cell cycle expression of the endogenously tagged TgMAPK-L1HA was revealed by co-staining with inner membrane protein (IMC1, red) and nuclear dye DAPI (blue). Strong expression of TgMAPK-L1 in the perinuclear region was detected in S-phase (S: second panel). After transient translocation to the newly forming daughter bud (M/C: third panel) TgMAPK-L1 gradually declined in cytokinesis (C: bottom panel). Cell cycle phases of individual parasites and vacuoles were determined based on well-established nuclear and cell morphological criteria [4]. (B) Genetic complementation of mutant 11–31G12 with cosmid genomic libraries identified a defective locus on the chromosome XI spanning three possible genes: TGGT1_312560 (gene 1), TGGT1_312570 (gene 2), and TGGT1_312580 (gene 3). Secondary complementation with cosmids spanning the locus (tiled cosmids) identified gene 3 encoding a putative CGMC kinase (TgMAPK-L1) as responsible for the ts-defect. The finding was further confirmed by complementation of mutant 11–31G12 with amplified genomic fragments (transformed cDNA) spanning the wild type or ts-allele. (C) Schematic of TgMAPK-L1 features shows the location of ts-mutation and the corresponding change in amino acid residue. (D) Strategy for introduction of the ts-TgMAPK-L1 mutation (L534Q) by genetic knock-in while simultaneously tagging the gene with 3xHA in the RHΔku80 strain. (E) ts-TgMAPK-L1HA (green) localized to the centrosomal region of dividing parasites (34°C) and in high temperature arrested parasites ts-TgMAPK-L1 was degraded (40°C). (F) Instability of the ts-TgMAPK-L1 at 40°C appears to be a major contributing factor to the growth arrest of mutant parasites at high temperature. Total lysates of RHΔku80 expressing ts-TgMAPK-L1HA grown for 24 h at 34°C or 40°C were analyzed by western blotting and probed with anti-HA antibody. Equal loading of parasite lysate was confirmed by counter stains with the anti-αTubulin antibody. (TIF) [file pbio.1002093.s005.tif]
