# Supplementary material for: A Novel Bipartite Centrosome Coordinates the Apicomplexan Cell Cycle
Source: PLoS Biol. 2015 Mar 3;13(3):e1002093. doi: 10.1371/journal.pbio.1002093 (PMC4348508; doi:10.1371/journal.pbio.1002093)
Supplement: S1 Table — (DOCX) [file pbio.1002093.s006.docx]

| **Gene ID**  **TGME49_** | **Product Description** | **Protein Length** | **e-value**  **(hCEP250)** | **Cell cycle stage** | **Localization** | **Comments** |
| --- | --- | --- | --- | --- | --- | --- |
| 212880 | surface antigen repeat protein | 6668 | 87 | N/A | **centrosome** | Current study: **TgCEP250** |
| 242750 | hypothetical | 3900 | 63 | N/A | unknown | Eliminated (not expressed in tachyzoite) |
| 254570 | hypothetical | 1972 | 58 | G1 | unknown | Current study: failed cloning* |
| 242790 | trichohyalin | 1761 | 50 | N/A | peripheral annuli | Current study: **TgPAP1** |
| 297210 | hypothetical | 3982 | 48 | G1/S | unknown | Current study: C-terminal tagging failed |
| 244470 | hypothetical | 2595 | 47 | S/M | apical cone | Gould et al., 2011 |
| 213392 | surface antigen repeat protein | 2315 | 42 | flat | unknown | Current study: C-terminal tagging failed |
| 239300 | hypothetical | 1232 | 39 | S/M | unknown | Eliminated (cell cycle stage) |
| 252880 | hypothetical | 3168 | 36 | M/C | conoid | Gould et al., 2011 |
| 290620 | hypothetical | 2662 | 36 | G1/S | **centrosome** | Current study: **TgCEP250L-1** |
| 257180 | RecF/RecN/SMC N-terminal domain protein | 1588 | 33 | G1 | unknown | Eliminated: predicted Rad50 |
| 265840 | hypothetical | 1338 | 31 | flat | nuclear envelope/pore | Current study: **TgNEP1** |

Two genomic loci resisted epitope tagging at the C-terminus of the corresponding protein (TGME49_297210 and TGME49_213392) and alone with futile cloning of TGME49_254570 resulted in elimination of these putative CEP proteins from further investigation.
